# A selective autophagy cargo receptor NBR1 modulates abscisic acid signalling in *Arabidopsis thaliana*

**DOI:** 10.1038/s41598-020-64765-z

**Published:** 2020-05-08

**Authors:** Leszek Tarnowski, Milagros Collados Rodriguez, Jerzy Brzywczy, Marta Piecho-Kabacik, Zuzana Krčkova, Jan Martinec, Anna Wawrzynska, Agnieszka Sirko

**Affiliations:** 1 Institute of Biochemistry and Biophysics Polish Academy of Sciences, ul. Pawinskiego 5A, 02-106, Warsaw, Poland; 2 Institute of Experimental Botany of the Czech Academy of Sciences, Rozvojova 263, 165 02, Prague 6, Czech Republic; 3 Present Address: MRC-University of Glasgow Center for Virus Research, 464 Bearsden Rd., Glasgow, G61 1QH Scotland United Kingdom

**Keywords:** Molecular biology, Plant sciences

## Abstract

The plant selective autophagy cargo receptor neighbour of breast cancer 1 gene (NBR1) has been scarcely studied in the context of abiotic stress. We wanted to expand this knowledge by using *Arabidopsis thaliana* lines with constitutive ectopic overexpression of the *AtNBR1* gene (OX lines) and the *AtNBR*1 Knock-Out (KO lines). Transcriptomic analysis of the shoots and roots of one representative OX line indicated differences in gene expression relative to the parental (WT) line. In shoots, many differentially expressed genes, either up- or down-regulated, were involved in responses to stimuli and stress. In roots the most significant difference was observed in a set of downregulated genes that is mainly related to translation and formation of ribonucleoprotein complexes. The link between AtNBR1 overexpression and abscisic acid (ABA) signalling was suggested by an interaction network analysis of these differentially expressed genes. Most hubs of this network were associated with ABA signalling. Although transcriptomic analysis suggested enhancement of ABA responses, ABA levels were unchanged in the OX shoots. Moreover, some of the phenotypes of the OX (delayed germination, increased number of closed stomata) and the KO lines (increased number of lateral root initiation sites) indicate that AtNBR1 is essential for fine-tuning of the ABA signalling pathway. The interaction of AtNBR1 with three regulatory proteins of ABA pathway (ABI3, ABI4 and ABI5) was observed *in planta*. It suggests that AtNBR1 might play role in maintaining the balance of ABA signalling by controlling their level and/or activity.

## Introduction

Autophagy is defined as a catabolic process responsible for degradation of cellular contents. Evolutionarily conserved, autophagy-related (ATG) proteins are organised in protein complexes to form an autophagosome (a double-membrane vesicle around the cargo) and manage its intracellular transport and fusion with the vacuole, where the cargo is degraded in plants. Autophagy is implicated in almost every aspect of plant growth, from embryogenesis to senescence, and in various stress responses, as recently reviewed^1–7^. Autophagy contributes to nutrient remobilisation during mineral starvation as well as during organ senescence *e.g*. remobilisation of nitrogen from the senescing leaves to seeds (see recent review^8^ and references therein). *Arabidopsis thaliana* autophagy-defective mutants are hypersensitive to carbon and nitrogen starvation, displaying early senescence even under nutrient-rich conditions (see recent review^9^ and references therein). Nutrient starvation and other abiotic stress conditions, such as heat, drought, saline and osmotic stress, oxidative stress, endoplasmic reticulum stress or sugar excess increase autophagic flux (see recent review^10^ and references therein).

Autophagy is tightly controlled to avoid excessive degradation of the cellular content. In normal conditions the process runs at a baseline level that increases when developmental and/or nutritional signals promote assembly of the ATG1/ATG13 autophagy initiation complex^11^. The formation of this complex is negatively regulated by the target of rapamycin (TOR) kinase. TOR acts as a master regulator of cellular and developmental processes. It is active under nutrient rich conditions, when it upregulates cell growth and translation whilst blocking autophagy, but it is inhibited during nutrient deficiency^12,13^. The plant TOR kinase complex is very similar in structure, mode of action and molecular function to its yeast and mammalian counterparts^14–17^. Comparative profiling of the transcriptomes of *tor* mutants and wild type (WT) plants indicated that TOR regulates photosynthesis^18^, the cell-cycle, cell-wall modifications, senescence, central energy metabolism, carbon and lipid metabolism and secondary metabolism^19^. The interplay of TOR with phytohormone signalling pathways is complex: it activates signalling by auxins, cytokines, brassinosteroids and gibberellin, whilst repressing signalling by abscisic acid (ABA), ethylene, jasmonic acid and salicylic acid^18,20,21^. Reciprocal regulation of TOR and ABA signalling to balance plant growth and stress responses has also been reported recently^22^.

Degradation of cellular content by autophagy can be highly selective. Autophagy cargo receptors ensure selective of autophagy by recognising a cargo tagged for degradation and docking it with the ATG8 protein anchored to the autophagosome^23–26^. Numerous cargo receptors and their cargos have been identified in metazoa, but only few have been characterised in plants, including neighbour of breast cancer 1 (NBR1). NBR1 binds protein aggregates and is involved in xenophagy^27,28^. In 2011 independent laboratories reported the presence of functional NBR1 receptors in two plant species, Arabidopsis^28^ and *Nicotiana tabaccum*^29^. Plant NBR1 proteins have several characteristic domains, PB1, which is involved in NBR1-NBR1 interaction, ZZ and NBR1/FW (both of unknown function) and the double UBA domain involved in ubiquitin (Ub) binding. It is unclear whether both UBA domains can interact with Ub. The tobacco protein (Joka2 or NtNBR1) is co-localises with actin, tubulin and ER^30^ and has also forms multimers via both PB1 and UBA domains^31^. Recent studies have concentrated on the role of NBR1 in plant defences against various pathogens^32–36^. In Arabidopsis, NBR1 is encoded at a single genetic locus. Studies with various insertional T-DNA mutants suggest that NBR1 is not essential. However, our detailed analysis of one such mutants indicated the presence of both the WT and the truncated transcripts, likely due to alternative splicing of the T-DNA insertion in the intron^37^. The ratio was variable and depended on growth conditions. Whether other insertional *nbr1* T-DNA mutants are affected by a similar phenomenon remains to be addressed.

Along with other plant hormones, abscisic acid (ABA) is involved in developmental processes: seed dormancy, plant growth, leaf senescence and response to environmental stresses^38^. The Ub–proteasome system regulates ABA perception and signalling by targeting ABA receptors, PP2C protein phosphatases, transcription factors and proteins encoded by ABA responsive genes. Post-translational control of ABA signalling involves numerous E3 ligases, kinases and phosphatases (see recent reviews^39,40^ and references therein). Links of autophagy and endomembrane trafficking with ABA signalling, synthesis and transport has also been established^41–43^. ABA signals are detected by 14 ABA receptors (PYR1 and PYL1–13, named also RCAR1–14). In the presence of ABA they interact with protein phosphatase 2 C (PP2C) family of phosphatases that downregulate ABA signalling. Nine PP2C phosphatases belonging to group A of the PP2C family are involved in regulation of the ABA pathway and are induced by ABA and stress. Multiple mutations in some of these genes elevate the ABA response^44^. In the absence of ABA, PP2Cs interact with SNF1-related protein kinase 2 (SnRK2) family of kinases and dephosphorylate them. Dephosphorylated SnRK2s are inactive, so this stops ABA signalling. Not only ABA signalling but also ABA production, transport and inactivation is tightly controlled. These aspects of ABA homeostasis were recently reviewed^45^. Essentially, ABA is synthesized de novo in multiple steps (by a series of enzymes) through the carotenoid pathway, while it is degraded mainly by a family of four ABA 8’-hydroxylases (CYP707A1–4) to phaseic acid (PA) and then, to dihydrophaseic acid (DPA)^46^. Essential role of these enzymes in ABA catabolism is highlighted by identification of numerous transcriptional factors inducing or repressing their expression (see review^45^ and references within). ABA homeostasis is also controlled by reversible glycosylation. The inactive ABA glucosyl ester can be stored in ER or in vacuole where it can be converted back to ABA due to the action of of specific glucosidases^47–49^. ABA transporters controlling its long-distance transport and movement across the plasma membrane are also crucial but not entirely characterized elements of ABA homeostasis and the regulatory components of the ABA-modulated processes^50^. ABA has important functions in all plant organs. For example in shoots ABA initiates the signalling cascade that closes stomata^51^, whilst in roots it regulates primary root growth by controlling division of the population of cells in the tip as well as regulating lateral root development by regulating initiation, emergence and meristem activation^52,53^. In addition, seed dormancy is maintained by ABA through ABI3 and ABI5 transcription factors which synergistically restrict embryo germination^54^.

This study focuses on the consequences of *AtNBR1* overexpression (OX) and disruption (KO) in Arabidopsis. Using a combinatorial approach, we conclude that the AtNBR1 autophagy cargo receptor is responsible for cross-talk between autophagy and the ABA signalling pathway. Overall, the excess or the lack of AtNBR1 protein enhances or reduces ABA signalling, respectively.

## Results

**Figure 1:**
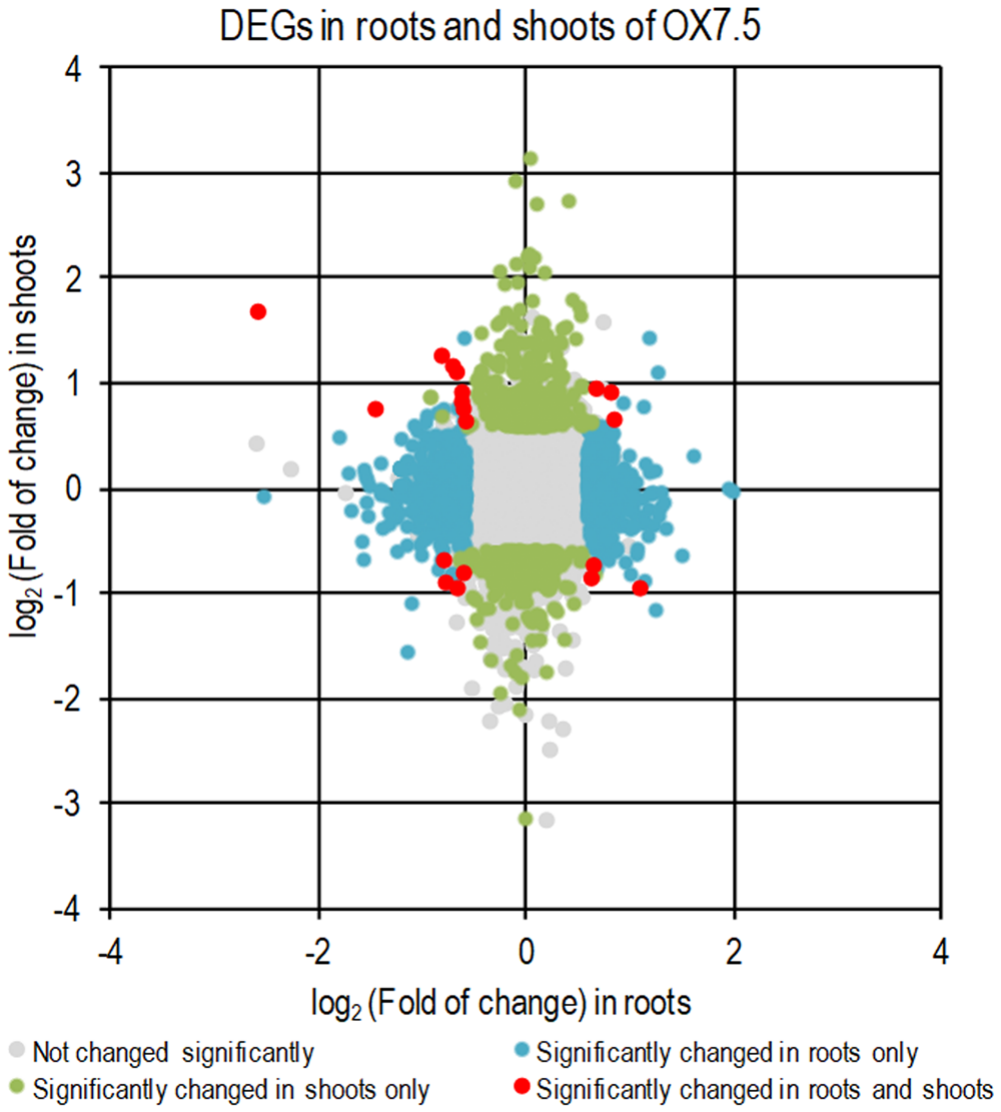
Differentially expressed genes (OX DEGs) in roots and shoots of OX7.5 in comparison to the wild type (WT).

AtNBR1 overexpression changes the gene expression profiles of both shoots and roots. Several NBR1-OX and nbr1-KO lines were obtained and verified by testing for the presence of the corresponding transcripts and proteins (Supplementary Fig. S1). A large set of differentially expressed genes (DEGs) were identified when the transcriptomes of 4-week-old OX7.5 and WT plants were compared, despite the lack of obvious morphological differences between the lines under the applied growth conditions (not shown). DEGs were defined as those with at least a 1.5-fold difference (p ≤ 0.05) relative to WT. In shoots, 294 of the 510 DEGs were upregulated, while 216 were downregulated (Supplementary Table S1), whereas in roots 416 of 919 DEGs were upregulated and 503 were downregulated (Supplementary Table S2). Remarkably, there were very few DEGs common to the two plant parts, prompting us to analyse the two data sets separately (Fig. 1). Among the GO categories associated with DEGs in shoots, ‘response to stress’ and ‘response to stimulus’ were over-represented among both up- and down-regulated genes, whereas ‘regulation of gene expression’, ‘transcription factor activity’, ‘DNA binding’ and ‘regulation of metabolic and cellular processes’ were only associated with genes upregulated in shoots (Supplementary Fig. S2). In roots, upregulated genes were associated with the categories ‘mitochondrion’ and ‘macromolecular complex’, whereas the categories ‘translation’, ‘gene expression’ and ‘nitrogen compound metabolic processes’ were strongly associated with downregulated genes (Supplementary Fig. S2). Thus, AtNBR1 overexpression affected shoots and roots differently and seems to influence multiple cellular processes and functions.

**Figure 2:**
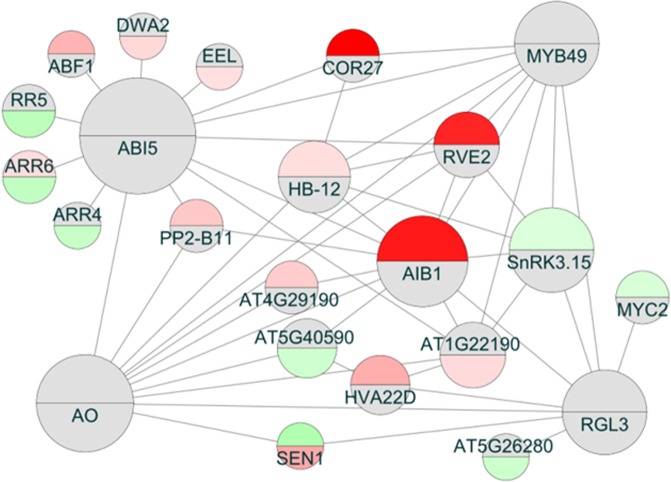
Interaction network of the OX DEGs. The putative network is composed of the regulatory hubs which had the highest numbers of DEGs associated with them (extracted with Cytoscape from the Biogrid network). Upregulated genes are shaded red and downregulated genes shaded green; shoots or roots are shown in the upper and lower parts of circles respectively). The gene symbols are explained in Supplementary Table S4 (Enriched Network spreadsheet).

**Figure 3:**
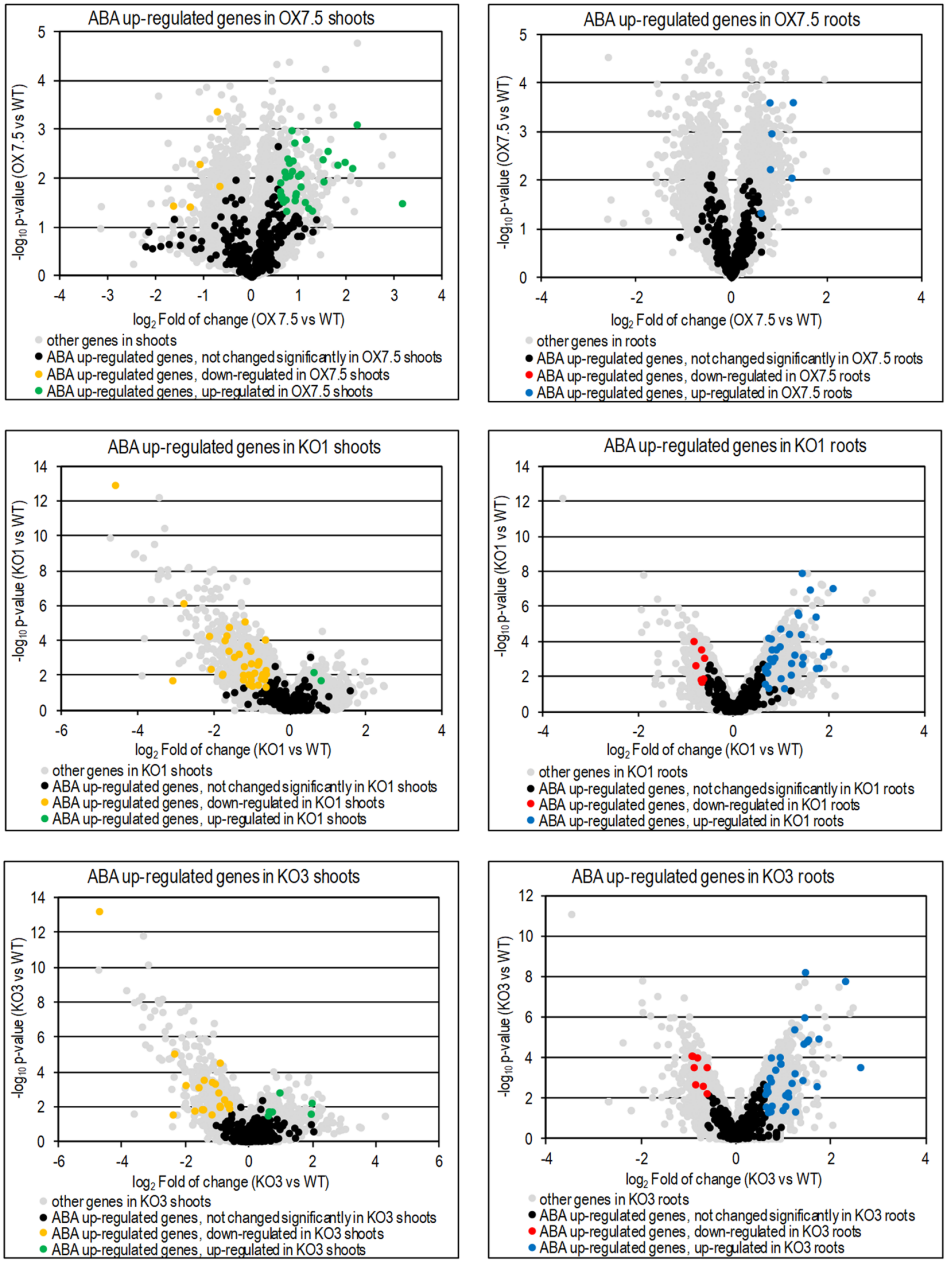
Changes of expression of ABA-upregulated genes in shoots and roots of OX7.5, KO1 and KO3 in comparison to WT.

In order to gain insight into the regulatory networks responsible for the observed changes in the transcriptome of the OX7.5 line overexpressing AtNBR1, we analysed known interactions of DEGs from the shoots and roots. Looking for putative regulatory hubs, we identified 64 nodes that interacted with at least 5 DEGs (Supplementary Table S3). However these hubs also had the highest total numbers of interactions, so we looked for hubs that were enriched in interactions with DEGs in order to identify hubs specifically involved in transcriptional responses to AtNBR1 overexpression. The selection was made on the basis of the ratio of the number of interacting DEGs to the total number of interacting nodes (Supplementary Table S4), and the calculated enrichment values were plotted against the number of interacting DEGs (Supplementary Fig. S3). This analysis identified the four hubs that were most enriched in interactions with DEGs: ABI5 (AT2G36270; bZIP transcription factor), RGL3 (AT5G17490; DELLA subfamily member), MYB49 (AT5G54230; MYB domain transcription factor) and AO (AT5G14760; aspartate oxidase catalysing the first key step of NAD+ biosynthesis). When the network of these four hubs was visualised along with interacting DEGs, two more putative regulatory hubs emerged: SR1 (AT5G01820; serine/threonine protein kinase 1, SnRK3.15; CIPK14) and AIB1 (AT1G10585; bHLH transcription factor) (Fig. 2). Both SR1 and AIB1 have been shown to be a part of the SnRK3-related abscisic acid (ABA) signalling network^55^. Transcript analysis revealed that many ABA upregulated genes^56^ were also upregulated by NBR1 overexpression, particularly in shoots (Fig. 3), whereas ABA downregulated genes were much less affected by NBR1 overexpression (Supplementary Fig. S4). It should be emphasised that although all DEGs were used for network construction, shoot DEGs were mainly responsible for the proposed association between AtNBR1 and ABA response, further indicating that AtNBR1 overexpression affects the shoot and root transcriptomes differently.

**Figure 4:**
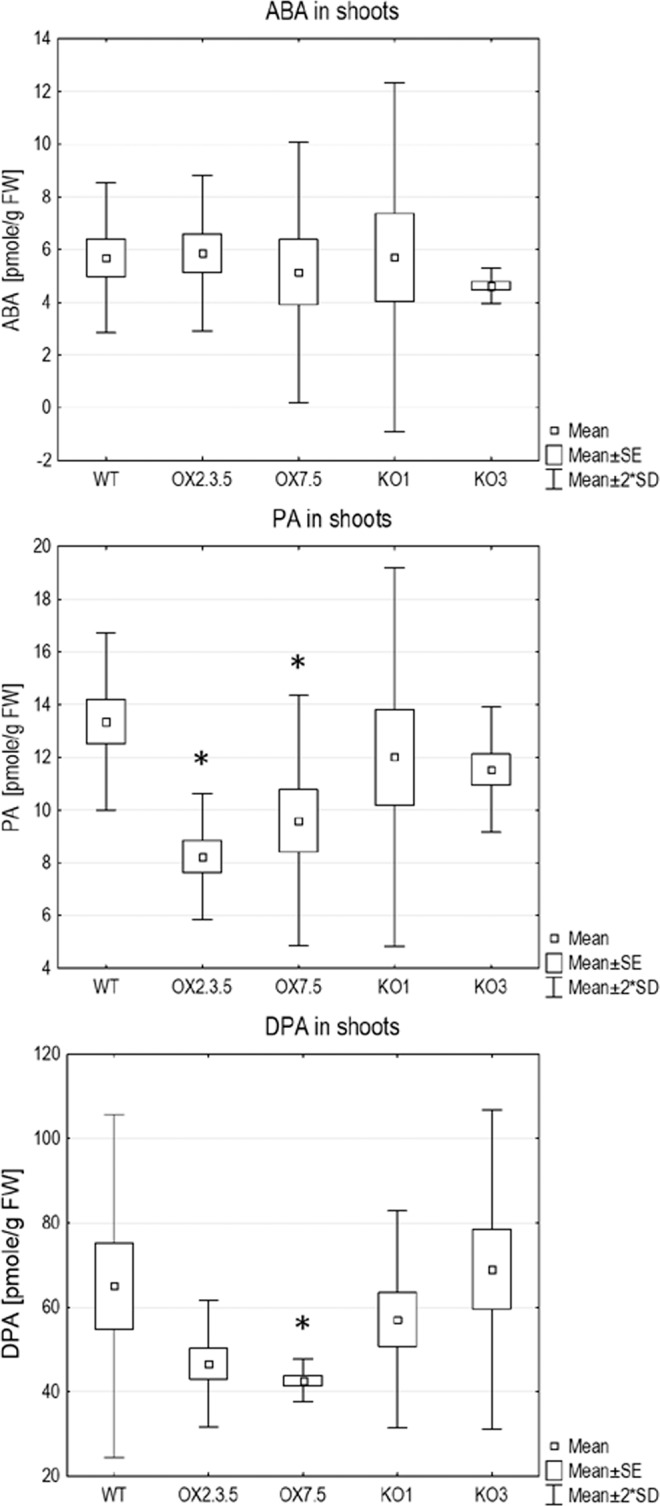
The level of abscisic acid (ABA), phaseic acid (PA) and dihydrophaseic acid (DPA) in shoots of WT, OX and KO lines. The asterisks indicate values significantly different (p < 0.05) from the WT. The difference was assessed using the Fisher’s LSD test (Supplementary Fig. S5).

Changes in expression of ABA-related genes in shoots and roots of OX7.5, KO1 and KO3 lines in comparison to the WT are shown in Supplementary Table S5. The expression of genes encoding enzymes involved in ABA biosynthesis or ABA catabolism was not changed significantly, except 4-fold downregulation of UGT71B7 in shoots of KO1. This result suggested that ABA metabolism was rather unaffected in OX and KO lines. To verify this conclusion, we assayed the level of endogenous ABA and its degradation products, PA and DPA in shoots of 4-week-old plants. The levels of PA and DPA were significantly lower in both OX lines than in the WT and KO lines, however, the levels of ABA were similar in all lines (Fig. 4, Supplementary Fig. S5). Therefore, we conclude that the gene expression changes observed in the shoots of OX7.5 did not results from increased ABA production or accumulation but from enhanced ABA sensing and/or signalling.

**Figure 5:**
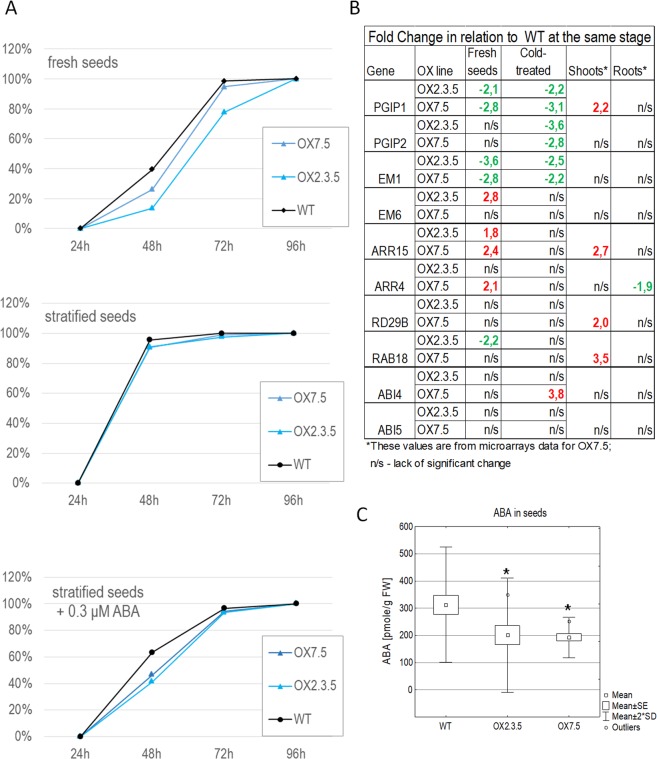
AtNBR1 overexpression modulates seed germination. (**A**) The results of one representative experiments are shown. Several independent experiments with different batches of seeds were conducted. The consistent differences in germination between the lines were observed in each experiment, however the percentages of germinated seeds varied, what hinders the statistical analysis of the germination results as an average from all experiments. (**B**) The relative levels of ABA-related transcripts in fresh and stratified WT and NBR1-OX seeds, assayed using RT-qPCR. (**C**) ABA level in the seeds. The asterisks indicate values significantly different (p < 0.05) from the WT. The difference was assessed using the Tukey’s HSD test (Supplementary Fig. S7).

Differences in seed germination suggest that OX lines have increased ABA sensitivity. Since ABA is known to regulate seed germination, we decided to check if the presumed enhanced ABA signalling in OX lines would have an impact on this process. Indeed, germination was delayed in OX seeds relative to WT seeds, but only in freshly collected batches of seeds (Fig. 5A). Such differences in germination disappeared when seeds were stored for as little as 10 days and when fresh seeds were kept at 4 ^o^C for 3–5 days before sowing. Besides, the OX lines were slightly more sensitive to ABA during germination. These results suggest an increase in seed dormancy due to AtNBR1 overexpression. Subsequently we monitored expression of several ABA related transcripts in fresh and cold-stratified seeds. We found that many of them were misregulated in both fresh and cold-stratified seeds of OX7.5 and OX2.3.5 lines (Fig. 5B, Supplementary Fig. S6). In some cases (*PGIP1*, *PGIP2*, *EM1*, *ARR15* and *ARR4*) the changes in gene expression were similar in the two OX lines, but in others the differences between the lines were quite substantial. For example, *ABI4* was similarly expressed in the WT and OX2.3.5 lines, but strongly enhanced in cold-stratified OX7.5 seeds. Since it was difficult to correlate the observed transcriptional changes with germination delays observed in fresh seeds from the OX lines, so the exact role of AtNBR1 during seed germination requires further investigation. Surprisingly, the stratified seeds from OX lines had lower level of ABA than those from WT (Fig. 5C, Supplementary Fig. 7). These results suggest that AtNBR1 overexpression enhances ABA signalling.

**Figure 6:**
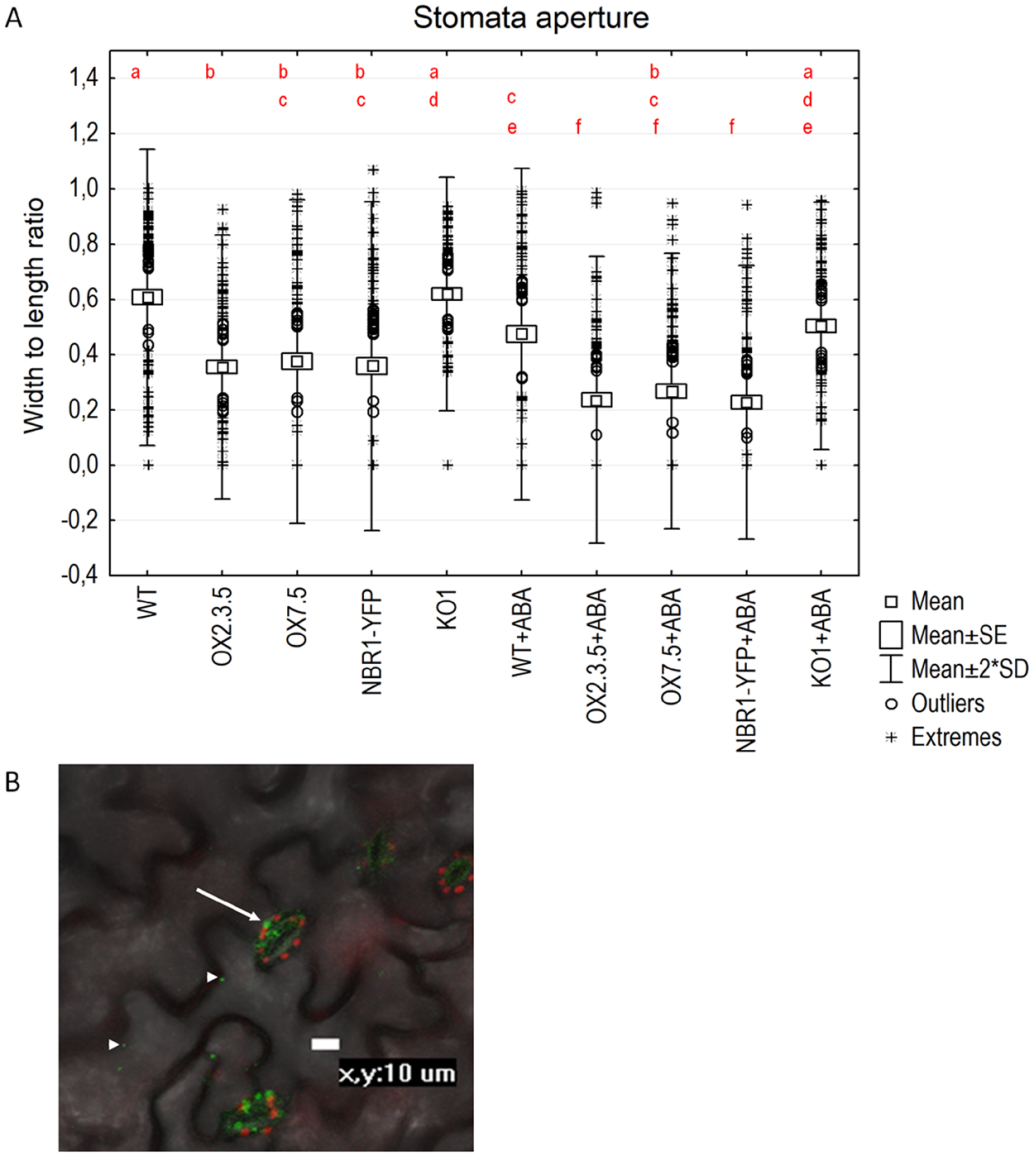
AtNBR1 overexpression reduces stomata aperture. (**A**)The results are calculated from 100 stomata per line per the treatment. The statistically significant differences are indicated by different letters. The difference was assessed using the Tukey’s HSD test (Supplementary Fig. S8). (**B**) Localisation of green fluorescence in transgenic *A. thaliana* seedlings overexpressing NBR1-YFP. Chloroplasts are shown in red. The arrows indicate strong signals in the stomata. The arrowheads indicate signals in the epidermal cells.

Reduction of stomatal opening in OX lines. Endogenous ABA is considered a main regulator of stomatal movement. Examination of stomatal aperture in seedlings in the absence of ABA indicated that the lines overexpressing AtNBR1 had significantly reduced stomatal aperture than the WT and KO lines (Fig. 6A, Supplementary Fig. S8). This result supports our assumption that NBR1 overexpression enhances ABA-related responses. Interestingly, the application of ABA stimulated stomatal closure in all investigated lines, however, the difference in the KO lines between the ABA-treated and non-treated material was statistically insignificant. These results suggest that NBR1 might be also involved in modulation of ABA-dependent stomata movement. In addition, a high concentration of yellow fluorescent protein (YFP) in guard cells of the transgenic line overexpressing *NBR1-YFP* points to an involvement of AtNBR1 in stomatal movement (Fig. 6B).

**Figure 7:**
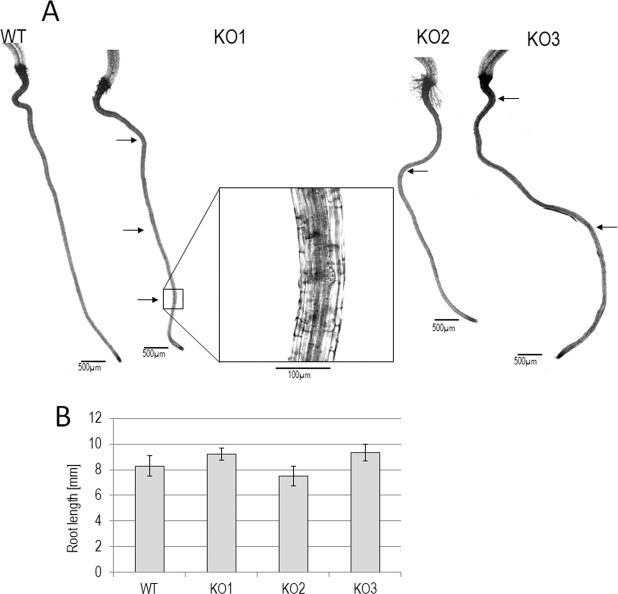
Lateral root initiation sites in 10-day-old seedlings grown in a liquid medium. (**A**) Typical examples of roots are shown with the position of the lateral root initiation sites marked with arrows. One site is shown at greater magnification to show details of the lateral root initiation site. The sites were present only in the KO lines. (**B**) The average length of the primary roots with standard deviations (SD) indicated (N = 5).

Enhanced lateral roots initiation in KO lines. The OX and KO lines did not show significant differences from the WT in primary roots growth in the absence or presence of 10 and 20 µM ABA (results not shown). However, some interesting differences were found in lateral root formation in 10-day-old seedlings grown in a hydroponic medium. Several sites of lateral root initiation (1–4 per root) were found in the KO lines, but no such sites were observed in the WT and OX lines. The location of initiation sites varied, with no apparent rule or preferred location (Fig. 7A). No differences between the lines were observed in this experiment with respect to primary root length (Fig. 7B), thus the increased formation of lateral roots in the nbr1-KO lines cannot be attributed to differences in the size or developmental stage of the primary roots.

### NBR1 interacts with regulators of ABA signalling

**Figure 8:**
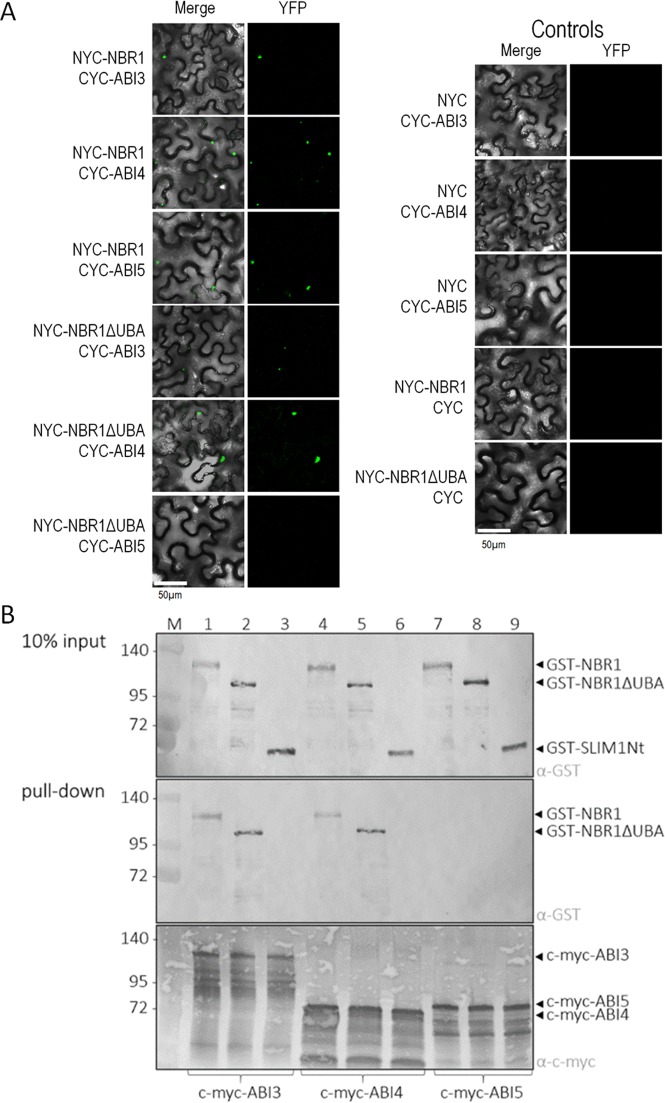
NBR1 interacts with ABI3, ABI4 and ABI5. (**A**) Results of BiFC experiments using full-length NBR1 or NBR1 with a C-terminal deletion of 87 amino acids (NBR1ΔUBA) and ABI3, ABI4 or ABI5. Interaction was considered to have taken place if a YFP signal is detected. (**B**) Pull-down assays using GST and c-myc-tagged proteins expressed in *E. coli*. Precipitated proteins were detected by immunoblotting. Input shows the presence of the GST-tagged proteins in crude extracts. After affinity purification through the anti-c-myc gel with attached ABI3, ABI4, or ABI5, the GST-tagged proteins NBR1 and NBR1ΔUBA (lanes 1, 4 and 2, 5, respectively) are detected due to interaction. There is no interaction detected between ABI5 and NBR1 nor NBRΔUBA (lanes 7 and 8, respectively). The negative controls, SLIM1Nt with the GST-tag are shown in lanes 3, 6 and 9. The molecular marker (M) with the size (kDa) of the proteins is indicated. Expected sizes of the fusion proteins: c-myc-ABI3, 109 kDa; c-myc-ABI4, 66 kDa; c-myc-ABI5, 77 kDa; GST-NBR1, 103 kDa; GST-NBR1ΔUBA, 93 kDa; GST-SLIM1Nt, 62 kDa. The primary antibodies recognizing the GST and myc tags are indicated as α-GST and α-myc, respectively.

The response to ABA is regulated by a complex network including transcriptional factors, of which the best known are ABI3, ABI4 and ABI5 (ABA-insensitive 3, 4 and 5, respectively), whose direct targets have already been identified^57,58^. Transcript analysis of genes directly regulated by ABI3, ABI4 and ABI5 indicated that only some of them were differently expressed in OX7.5; based on these results it is impossible to identify the (single) factor responsible for the gene expression changes in OX7.5. Given that AtNBR1 acts as a selective autophagy cargo receptor it seemed probable that it would interact physically with some transcriptional regulators of ABA signalling. We therefore tested whether ABI3, ABI4 and ABI5, interact with AtNBR1 in a bimolecular fluorescence complementation (BiFC) *in planta* experiment. Indeed, AtNBR1 can interact with all of them. Interestingly, binding to ABI3 and ABI4, in contrast to ABI5 binding, did not require the ubiquitin-associated (UBA) domain of AtNBR1 (Fig. 8A) that is involved in binding to Ub-tagged proteins^28^. This implies that binding of NBR1 to ABI5 probably requires prior ubiquitination of ABI5, whereas binding of NBR1 to ABI3 and ABI4 appears to be independent of ubiquitination. The binding of ABI3 and ABI4 to both forms of NBR1 (full-length NBR1 and NBR1ΔUBA) was confirmed by the pull-down assay using the recombinant proteins purified from *E.coli* cells (Fig. 8B, Fig. S9). The pull-down assay did not confirm the binding of ABI5 to NBR1 highlighting the importance of potential ubiquitination for such interaction. Further clarification of the significance and molecular details of the NBR1-ABI3, NBR1-ABI4 and NBR1-ABI5 interactions and the physical binding of NBR1 to other proteins of the ABA signal transduction pathway is required.

## Discussion

The aim of this study was to characterise the NBR1-OX lines and compare their leaf and root transcriptomes with those of the WT. We report for the first time the existence of crosstalk between AtNBR1 and ABA signalling (Fig. 2). Two hubs of the interaction network of the differentially expressed genes (DEGs) in OX7.5 are involved in ABA signalling pathway: (i) ABI5 (AT2G36270, bZIP TF), which is the main regulator of ABA signalling and is also involved in controlling seed dormancy and germination^38,59^ and (ii) AIB1 (AT1G10585, bHLH TF), which has been identified as an element of the SnRK3 (SnRK3.15/SnRK3.22) network and as a central hub of the ABA-regulated transcriptional network^55^. In fact, other elements of the SNRK3 network were also identified as hubs in our interaction network: SR1 (AT5G01820, SnRK3.15), RGL3 (AT5G17490, DELLA family), and MYB49 (AT5G54230). RGL3 integrates ABA, auxin and gibberellin (GA) signalling^60–63^, whilst MYB49 has recently been linked with cadmium resistance^64^.

We then examined the potential effects of NBR1 overexpression on ABA-regulated processes, such as seed germination and regulation of stomata aperture and root growth. Delayed germination of OX lines’ fresh seeds (Fig. 5) could be attributed to their enhanced ABA sensitivity, despite displaying lower amount of ABA in the seeds (Fig. 5C). Stomatal closure was also significantly enhanced in three independent AtNBR1 overexpressors (Fig. 6A). In line with the positive role of AtNBR1in ABA signalling, the reduced expression of several ABA-up regulated genes were observed in shoots of two KO lines (Fig. 3). Moreover, the careful microscopic investigation of the roots of seedlings grown in a hydroponic medium indicated the presence of several lateral root initiation sites (from 1 to 4 per plantlet) in all the seedlings from all three tested deletions lines (KO1-3) (Fig. 7). In contrast, such sites were almost absent from WT and OX seedlings grown under the same conditions (liquid medium). These results, along with the lack of evidence for an increase in ABA level in the shoots of OX lines, strongly suggest that AtNBR1 is involved in modulating ABA signalling. The OX lines showed enhanced ABA sensitivity, whereas ABA signalling seems to be reduced in KO lines, however it is yet unclear which stages of ABA regulation pathway are modulated by NBR1.

Published data suggest that complex interplay among regulatory factors is responsible for the tested phenotypes. The main regulators, ABI3, ABI4 and ABI5, have been linked to seed dormancy^54,65,66^, stomata closure^67^ and initiation and growth of lateral roots^68,69^. Although the details of how AtNBR1 is involved in ABA signalling remain to be determined, the finding that AtNBR1 is capable of binding to ABI3, ABI4 and ABI5 *in planta* is the first report of such interaction (Fig. 8). It is unclear whether AtNBR1 affects the stability or function of these proteins and if it targets them at autophagosomes. ABI5 binds only a full-length AtNBR1, whereas ABI3 and ABI4 also binds truncated AtNBR1 (without UBA domains; NBR1ΔUBA), which suggests that ABI3 and ABI4 may not need to be ubiquitinated in order to interact with AtNBR1; it is possible that a different type of interaction from the classical ‘ubiquitinated target - autophagy receptor’ is involved. Despite of the interaction of AtNBR1 with ABI3, ABI4 and ABI5, we failed to detect significant changes of expression of direct targets of ABI3, ABI4 or ABI5 that had been identified before^57,58^. Instead, interaction network analysis indicated that the AIB1 (AT1G10585) and SnRK3 networks act as important for regulation of the OX DEGs. It is also possible that AtNBR1 acts as an autophagy receptor for other elements of the ABA pathway, for example it could stimulate autophagic degradation of some of the PP2C phosphatases that act as negative regulators of ABA signalling^40^.

**Figure 9:**
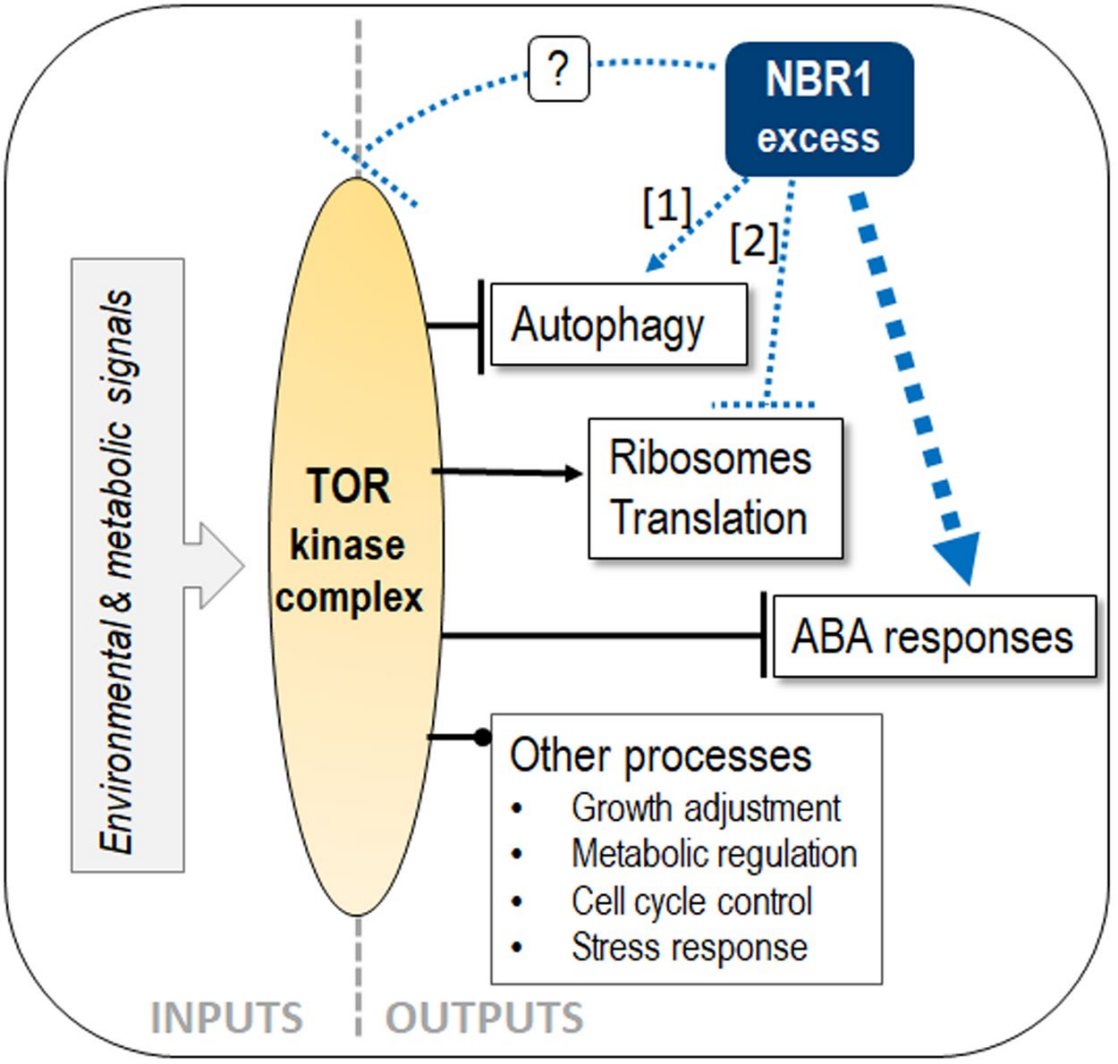
Hypothetical model of NBR1 involvement in balancing ABA and TOR signalling pathways in plants. TOR complex negatively regulates autophagy and ABA signalling. Excess of NBR1 can stimulate ABA signalling via modulation of the ABA pathway (blue thick arrow), possibly increasing the number of autophagosomes [1], modulating ribosomes function or translation [2], however, it might also act indirectly, by inhibiting the TOR activity (dotted line with question mark).

In summary, our results indicate that OX lines are oversensitive to ABA. Based on the observed interaction between AtNBR1 and such ABA-related transcription factors as ABI3, ABI4 and ABI5, it is tempting to speculate that AtNBR1 is involved in maintaining the balance of the regulatory elements involved in ABA signalling, however, we cannot exclude that it could also fine tune other key elements of ABA sensing/signalling pathway. In a hypothetical model we propose that the observed enhancement of ABA signalling in the OX lines is due to direct modulation of the ABA pathway by AtNBR1 (Fig. 9). However, our recent results suggest that AtNBR1 overexpression enhances autophagy and modulates ribosome function^70^. Therefore, we cannot exclude that AtNBR1 overexpression can also modulate other aspects of TOR signalling, acting upstream or downstream of TOR, or at both levels.

## Materials and methods

### Plant material

The plant lines used in this study are described in Table 1 and Supplementary Fig. S1.

**Table 1:** Arabidopsis thaliana lines used in this study. All transgenic lines were generated by *Agrobacterium*-mediated genetic transformation of the WT.

Line	Description (resistance marker in plants)	Source
Col-0 (WT)	Parental line	NASC, Nottingham, UK
OX2.3.5	Constitutive overexpression of AtNBR1-TAP. Expression cassette was generated using the binary vector pYL436^78^. Transgene expression was positively verified in OX2.3.5 and OX7.5 but not in OX3.5 and OX4.5 (gentamycin)	This work; for more details see Fig. S1
OX3.5		
OX4.5		
OX7.5		
NBR1-YFP	Constitutive overexpression of AtNBR1-YFP. Expression cassette was generated using the binary vector pH7YWG2^79^ (hygromycin)	This work;
nbr1-KO1 (KO1) nbr1-KO2 (KO2) nbr1-KO3 (KO3)	Deletion in NBR1 gene were generated using the CRISPR/Cas9 method^80^. The genomic regions covering the deletions were amplified and sequenced. The transgenic inserts were crossed-out from the homozygous KO	This work; see also Supplementary Fig. S1E and S1F

### Plant growth conditions

The composition of the plant media, 0.5 x Hoagland (used in most experiments) and 0.5 x AB (used only for the microarray experiment with OX line) is provided in Supplementary material (Table S6). Seeds were dry sterilised as described previously^71^ and placed on the solid medium with 0.8% Agargel (Sigma-Aldrich Sp. z o.o., Poland). All seeds were usually stratified (4 days, 4 ^o^C) unless specified differently, and maintained in a growth chamber. Germinated seeds were counted in the indicated time (24, 48, 72 and 96 h) after sowing. A seed was considered to have germinated when the radicle had ruptured the endosperm and testa. Germination experiments were repeated 3–4 times, with 20–30 seeds from each line per condition, using independent batches of seeds. The seedlings and plants used for microscopic observations and for collection of shoot and root tissue were usually grown in hydroponic condition. Plant material for transcript profiling was grown in Araponics boxes (Araponics, Belgium) (16 plants/box) kept in short day conditions (22 °C 8 h day/18 °C 16 h night) for about 4 weeks, with weekly changes of medium.

Isolation of RNA. Individual plants were divided into shoot and root tissue and frozen in liquid nitrogen. Portions of plant material, drawn from at least 3 plants, were homogenised in TRI Reagent (Molecular Research Centre, Cincinnati, OH, USA), and genomic DNA was removed with a RapidOut DNA removal kit (Thermo Fisher Scientific, Waltham, MA, USA). The quality, integrity and the concentration of RNA were evaluated using a Bioanalyzer 2100 (Agilent Technologies, Sana Clara, CA, USA) and a Nano Drop ND-100 (Thermo Fisher Scientific, Waltham, MA, USA) spectrophotometer. After the quality of the RNA preparations had been checked, cDNA was obtained by reverse transcription and used for real-time quantitative polymerase chain reactions (qPCR) and/or microarray analysis.

### Microarray analysis

Samples containing 100 ng total RNA that had passed the initial quality control test were reverse transcribed using the Ambion WT Expression Kit and labelled with the Affymetrix GeneChip WT Terminal Labelling Kit (Thermo Fisher Scientific, Waltham, MA, USA), according to the manufacturers’ protocols. Labelled cDNA probes were hybridised to the Affymetrix Arabidopsis Gene 1.1 ST Array Strips (Thermo Fisher Scientific, Waltham, MA, USA). Three independent biological replication of shoots and roots were analysed using the Affymetrix Gene Atlas system at the Laboratory of Microarray Analysis, Institute of Biochemistry and Biophysics of the Polish Academy of Sciences. Raw data were normalised and subjected to a three-way ANOVA to create lists of genes expressed differently in the biological variants (fold changes of at least ±1.5, p < 0.05).

### Oligonucleotides and plasmids

The list of oligonucleotides used as primers is provided in Supplementary Table S7. The list of plasmids used in this study is provided in Supplementary Table S8.

### Real-time quantitative polymerase chain reactions

The quantitative PCR (qPCR) was performed in a PikoReal Real-Time PCR System (Thermo Fisher Scientific, Waltham, MA, USA) with Luminaris Color HiGreen qPCR Master Mix (Thermo Fisher Scientific). Actin 2 (At3g18780), a constitutively expressed gene, was used to normalise the quantity of total RNA present in each sample. Relative gene expression levels were calculated using the delta-delta Ct method. Operator variance was assessed using qPCR in 3 biological replicates, each in triplicate.

### ABA assay

ABA level in seeds was assayed according to a previously published procedure^72^, using the Phytodetek ABA Test Kit (Agdia, Elkhart, IN, USA). The assay was done in 3 biological replicates, each performed as 3 technical replicates. ABA, PA and DPA level in shoots was assayed using the liquid chromatography coupled to mass spectrometry (LC-MS) method. The samples were flash frozen in liquid nitrogen. Plant hormones were extracted, purified and quantified according to reported procedures^73,74^. Briefly, samples were extracted with extraction buffer methanol/H_2_O/formic acid (15:4:1, v-v:v) supplemented with stable isotope-labelled internal standards (10 pmol per sample). The extracts were purified by solid phase extraction (SPE) using Oasis MCX cartridges (Waters, Milford, MA, USA). The SPE eluates were evaporated to dryness and pellets dissolved in 30 μl of 5% methanol in water (v/v). Quantification was done on an Ultimate 3000 high-performance liquid chromatograph (HPLC; Dionex, Bannockburn, IL, USA) coupled to a 3200 Q TRAP hybrid triple quadrupole/linear ion trap mass spectrometer (Applied Biosystems, Foster City, CA, USA).) using isotope dilution method. Data processing was carried out with Analyst 1.5 software (Applied Biosystems).

### Confocal microscopy

All observations were made using a Eclipse TE2000-E inverted confocal microscope (Nicon Instruments Inc., New Yourk, NY, USA). Acidic speckles (such as autophagosmomes) were stained for 15 min with 1 μg/ml acridine orange (Invitrogen), and observed using a 543 nm laser (helium-neon laser; Melles Griot, NY, USA) and 605/75 filter. Stomata were observed in visual light and counted using the saved photographs. Figures for stomatal aperture are based on results from at least 3 independent seedlings (at least 5 slides per plant). The leaves used to assess stomata were floated on the buffer (50 mM KCl, 0.1 mM CaCl_2_, 10 mM MES pH6.5) for 90 min. To assess the influence of ABA on stomata aperture the leaves were then transferred to buffer supplemented with 10 µM ABA for an additional 90 min. Fluorescence from NBR1-YFP was observed in fresh leaves using a 488 nm laser (Sapphire 488–20 CDRH, Coherent, Santa Clara, CA, USA) and 515/30 filter. Bimolecular fluorescence complementation (BiFC), used to test protein-protein interactions in plants, was monitored 2 days after agroinfiltration of *Nicotiana benthamiana* leaves with *Agrobacterium tumefaciens* cells (strain GV3101) transformed with the indicated combination of plasmids. The plasmids encoded the N-terminal (pSITE-nEYFP-C1) or C-terminal (pSITE-cEYFP-C1) part of YFP^75^ fused to the proteins or protein fragments being investigated. Bacteria were grown for 24 h at 28 °C in YEB medium supplemented with 10 μg/ml rifampicin and 50 μg/ml spectinomycin prior to agroinfiltration. Interactions were tested using a 488 nm laser (Sapphire 488–20 CDRH, Coherent, Santa Clara, CA, USA) and 515/30 filter. Seedlings for observation of lateral roots were stained with 0.05% Ruthenium red for 8 min and washed twice in H_2_O.

### Pull-down experiments

The plasmids encoding the N-terminal GST or c-myc tag linked to the proteins being investigated are listed in Supplementary Table S8. All GST and c-myc-tagged proteins were expressed in *Escherichia coli* Rosetta (DE3). Total proteins containing c-myc fusion proteins (ABI3, ABI4, ABI5) were extracted from bacteria by sonication in RIPA buffer (ThermoFisher Scientific, Waltham, MA, USA) supplemented with Proteinase Inhibitor Cocktail (Sigma-Aldrich Sp. z o.o., Poland) and purified on EZview Red Anti-c-myc Affinity Gel (Sigma-Aldrich Sp. z o.o., Poland) according to manufacturer’s protocol. Next, the crude extracts containing GST fusion proteins (NBR1, NBR1ΔUBA and control protein SLIM1Nt) were incubated with above prepared raisins in 4 °C overnight with gentle rocking. The raisins were washed 6 times with RIPA buffer, boiled with 2x SDS-PAGE gel loading buffer, subjected to SDS-PAGE and next immunoblotted using mouse monoclonal anti-GST IgG and anti-c-myc IgG (Sigma-Aldrich Sp. z o.o., Poland; Cat. No. G1160 and Santa Cruz Biotechnology Inc. Dallas, TX, USA; Cat. No. SC-40) as primary antibody and anti-mouse IgG conjugated to alkaline phosphatase (Sigma-Aldrich Sp. z o.o., Poland; Cat. No. A3562) as secondary antibody.

### Bioinformatic tools

The statistical significance of group differences was assessed with the Fisher’s Least Significant Difference (LSD) test of ANOVA or Tukey’s Honest Significant Difference (HSD) test, using Statistica 12 software (StatSoft Polska Sp. z o.o., Poland). Differences were considered significant at p < 0.05. Function was ascribed to the obtained lists of genes using the AgriGO v2.0 analysis tool [http://systemsbiology.cau.edu.cn/agriGOv2/] with TAIR10_2017 background and Plant GO Slim list of GO terms. Interaction networks were analysed with Cytoscape (ver. 3.7.1) software^76^ and visualised using the enhanced Graphics (ver. 1.2.0) plugin^77^.

## Supplementary information

Supplementary Information.

Supplementary Information.

## Data Availability

The transcriptomic data generated in this study are accessible through GEO Series accession numbers GSE122705 and GSE147124.

## References

[1] Batoko H, Dagdas Y, Baluska F, Sirko A (2017). Understanding and exploiting autophagy signaling in plants. Essays Biochem..

[2] Galluzzi L (2017). Molecular definitions of autophagy and related processes. EMBO J..

[3] Jacomin AC, Gul L, Sudhakar P, Korcsmaros T, Nezis IP (2018). What We Learned From Big Data for Autophagy Research. Front. Cell Dev. Biol..

[4] Liu F, Marshall RS, Li F (2018). Understanding and exploiting the roles of autophagy in plants through multi-omics approaches. Plant. Sci..

[5] Marshall RS, Vierstra RD (2018). Autophagy: The Master of Bulk and Selective Recycling. Annu. Rev. Plant. Biol..

[6] Wang P, Mugume Y, Bassham DC (2018). New advances in autophagy in plants: Regulation, selectivity and function. Semin. Cell Dev. Biol..

[7] Zientara-Rytter K, Sirko A (2016). To deliver or to degrade - an interplay of the ubiquitin-proteasome system, autophagy and vesicular transport in plants. FEBS J..

[8] Masclaux-Daubresse C, Chen Q, Have M (2017). Regulation of nutrient recycling via autophagy. Curr. Opin. Plant. Biol..

[9] Thompson AR, Doelling JH, Suttangkakul A, Vierstra RD (2005). Autophagic nutrient recycling in Arabidopsis directed by the ATG8 and ATG12 conjugation pathways. Plant. Physiol..

[10] Signorelli S, Tarkowski LP, Van den Ende W, Bassham DC (2019). Linking Autophagy to Abiotic and Biotic Stress Responses. Trends Plant. Sci..

[11] Suttangkakul A, Li F, Chung T, Vierstra RD (2011). The ATG1/ATG13 protein kinase complex is both a regulator and a target of autophagic recycling in Arabidopsis. Plant. Cell.

[12] Gonzalez A, Hall MN (2017). Nutrient sensing and TOR signaling in yeast and mammals. EMBO J..

[13] Robaglia C, Thomas M, Meyer C (2012). Sensing nutrient and energy status by SnRK1 and TOR kinases. Curr. Opin. Plant. Biol..

[14] Rexin D, Meyer C, Robaglia C, Veit B (2015). TOR signalling in plants. Biochem. J..

[15] Shi, L., Wu, Y. & Sheen, J. TOR signaling in plants: conservation and innovation. Development 145, 10.1242/dev.160887 (2018). 10.1242/dev.160887 PMC6053665 29986898

[16] Xiong Y, Sheen J (2015). Novel links in the plant TOR kinase signaling network. Curr. Opin. Plant. Biol..

[17] Saxton RA, Sabatini DM (2017). mTOR Signaling in Growth, Metabolism, and Disease. Cell.

[18] Dong P (2015). Expression profiling and functional analysis reveals that TOR is a key player in regulating photosynthesis and phytohormone signaling pathways in Arabidopsis. Front. Plant. Sci..

[19] Caldana C (2013). Systemic analysis of inducible target of rapamycin mutants reveal a general metabolic switch controlling growth in Arabidopsis thaliana. Plant. J..

[20] Salem MA (2018). RAPTOR Controls Developmental Growth Transitions by Altering the Hormonal and Metabolic Balance. Plant. Physiol..

[21] Kravchenko A (2015). Mutations in the Arabidopsis Lst8 and Raptor genes encoding partners of the TOR complex, or inhibition of TOR activity decrease abscisic acid (ABA) synthesis. Biochem. Biophys. Res. Commun..

[22] Wang P (2018). Reciprocal Regulation of the TOR Kinase and ABA Receptor Balances Plant Growth and Stress Response. Mol. Cell.

[23] Kim BW, Kwon DH, Song HK (2016). Structure biology of selective autophagy receptors. BMB Rep..

[24] Stolz A, Ernst A, Dikic I (2014). Cargo recognition and trafficking in selective autophagy. Nat. Cell Biol..

[25] Zaffagnini G, Martens S (2016). Mechanisms of Selective Autophagy. J. Mol. Biol..

[26] Kirkin, V. History of The Selective Autophagy Research: How Did It Begin and Where Does It Stand Today? J Mol Biol, 10.1016/j.jmb.2019.05.010 (2019). 10.1016/j.jmb.2019.05.010 PMC6971693 31082435

[27] Zhou J (2013). NBR1-mediated selective autophagy targets insoluble ubiquitinated protein aggregates in plant stress responses. PLoS Genet..

[28] Svenning S, Lamark T, Krause K, Johansen T (2011). Plant NBR1 is a selective autophagy substrate and a functional hybrid of the mammalian autophagic adapters NBR1 and p62/SQSTM1. Autophagy.

[29] Zientara-Rytter K (2011). Identification and functional analysis of Joka2, a tobacco member of the family of selective autophagy cargo receptors. Autophagy.

[30] Zientara-Rytter K, Sirko A (2014). Selective autophagy receptor Joka2 co-localizes with cytoskeleton in plant cells. Plant. Signal. Behav..

[31] Zientara-Rytter K, Sirko A (2014). Significant role of PB1 and UBA domains in multimerization of Joka2, a selective autophagy cargo receptor from tobacco. Front. Plant. Sci..

[32] Dagdas, Y. F. *et al*. An effector of the Irish potato famine pathogen antagonizes a host autophagy cargo receptor. Elife 5, 10.7554/eLife.10856 (2016). 10.7554/eLife.10856 PMC4775223 26765567

[33] Dagdas, Y. F. *et al*. Host autophagy machinery is diverted to the pathogen interface to mediate focal defense responses against the Irish potato famine pathogen. Elife 7, 10.7554/eLife.37476 (2018). 10.7554/eLife.37476 PMC6029844 29932422

[34] Hafren A, Hofius D (2017). NBR1-mediated antiviral xenophagy in plant immunity. Autophagy.

[35] Hafren A (2017). Selective autophagy limits cauliflower mosaic virus infection by NBR1-mediated targeting of viral capsid protein and particles. Proc. Natl Acad. Sci. USA.

[36] Hafren A (2018). Turnip Mosaic Virus Counteracts Selective Autophagy of the Viral Silencing Suppressor HCpro. Plant. Physiol..

[37] Rodriguez MC, Wawrzynska A, Sirko A (2014). Intronic T-DNA insertion in Arabidopsis NBR1 conditionally affects wild-type transcript level. Plant. Signal. Behav..

[38] Yoshida T, Mogami J, Yamaguchi-Shinozaki K (2015). Omics Approaches Toward Defining the Comprehensive Abscisic Acid Signaling Network in Plants. Plant. Cell Physiol..

[39] Yu F, Wu Y, Xie Q (2016). Ubiquitin-Proteasome System in ABA Signaling: From Perception to Action. Mol. Plant..

[40] Yang W, Zhang W, Wang X (2017). Post-translational control of ABA signalling: the roles of protein phosphorylation and ubiquitination. Plant. Biotechnol. J..

[41] Yu F (2016). ESCRT-I Component VPS23A Affects ABA Signaling by Recognizing ABA Receptors for Endosomal Degradation. Mol. Plant..

[42] Yu F, Xie Q (2017). Non-26S Proteasome Endomembrane Trafficking Pathways in ABA Signaling. Trends Plant. Sci..

[43] Vanhee C, Zapotoczny G, Masquelier D, Ghislain M, Batoko H (2011). The Arabidopsis multistress regulator TSPO is a heme binding membrane protein and a potential scavenger of porphyrins via an autophagy-dependent degradation mechanism. Plant. Cell.

[44] Antoni R (2012). Selective inhibition of clade A phosphatases type 2C by PYR/PYL/RCAR abscisic acid receptors. Plant. Physiol..

[45] Ma, Y. *et al*. Molecular Mechanism for the Regulation of ABA Homeostasis During Plant Development and Stress Responses. Int J Mol Sci 19, 10.3390/ijms19113643 (2018). 10.3390/ijms19113643 PMC6274696 30463231

[46] Saito S (2004). Arabidopsis CYP707As encode (+)-abscisic acid 8′-hydroxylase, a key enzyme in the oxidative catabolism of abscisic acid. Plant. Physiol..

[47] Priest DM (2006). Use of the glucosyltransferase UGT71B6 to disturb abscisic acid homeostasis in Arabidopsis thaliana. Plant. J..

[48] Lee KH (2006). Activation of glucosidase via stress-induced polymerization rapidly increases active pools of abscisic acid. Cell.

[49] Xu ZY (2012). A vacuolar beta-glucosidase homolog that possesses glucose-conjugated abscisic acid hydrolyzing activity plays an important role in osmotic stress responses in Arabidopsis. Plant. Cell.

[50] Kuromori T, Seo M, Shinozaki K (2018). ABA Transport and Plant Water Stress Responses. Trends Plant. Sci..

[51] Yoshida, T. *et al*. Insights into ABA-mediated regulation of guard cell primary metabolism revealed by systems biology approaches. Prog Biophys Mol Biol, 10.1016/j.pbiomolbio.2018.11.006 (2018). 10.1016/j.pbiomolbio.2018.11.006 30447225

[52] Harris JM (2015). Abscisic Acid: Hidden Architect of Root System Structure. Plants.

[53] Zhang H (2010). ABA promotes quiescence of the quiescent centre and suppresses stem cell differentiation in the Arabidopsis primary root meristem. Plant. J..

[54] Lopez-Molina L, Mongrand S, McLachlin DT, Chait BT, Chua NH (2002). ABI5 acts downstream of ABI3 to execute an ABA-dependent growth arrest during germination. Plant. J..

[55] Lumba S (2014). A mesoscale abscisic acid hormone interactome reveals a dynamic signaling landscape in Arabidopsis. Dev. Cell.

[56] Goda H (2008). The AtGenExpress hormone and chemical treatment data set: experimental design, data evaluation, model data analysis and data access. Plant. J..

[57] Monke G (2012). Toward the identification and regulation of the Arabidopsis thaliana ABI3 regulon. Nucleic Acids Res..

[58] Reeves WM, Lynch TJ, Mobin R, Finkelstein RR (2011). Direct targets of the transcription factors ABA-Insensitive(ABI)4 and ABI5 reveal synergistic action by ABI4 and several bZIP ABA response factors. Plant. Mol. Biol..

[59] Skubacz A, Daszkowska-Golec A, Szarejko I (2016). The Role and Regulation of ABI5 (ABA-Insensitive 5) in Plant Development, Abiotic Stress Responses and Phytohormone Crosstalk. Front. Plant. Sci..

[60] Piskurewicz U, Lopez-Molina L (2009). The GA-signaling repressor RGL3 represses testa rupture in response to changes in GA and ABA levels. Plant. Signal. Behav..

[61] Shi H, Liu W, Wei Y, Ye T (2017). Integration of auxin/indole-3-acetic acid 17 and RGA-LIKE3 confers salt stress resistance through stabilization by nitric oxide in Arabidopsis. J. Exp. Bot..

[62] Shu K, Zhou W, Chen F, Luo X (2018). & Yang, W. Abscisic Acid and Gibberellins Antagonistically Mediate Plant Development and Abiotic Stress Responses. Front. Plant. Sci..

[63] Shu K, Zhou W, Yang W (2018). APETALA 2-domain-containing transcription factors: focusing on abscisic acid and gibberellins antagonism. N. Phytol..

[64] Zhang P (2019). The R2R3-MYB Transcription Factor MYB49 Regulates Cadmium Accumulation. Plant. Physiol..

[65] Chen C (2014). ASCORBATE PEROXIDASE6 protects Arabidopsis desiccating and germinating seeds from stress and mediates cross talk between reactive oxygen species, abscisic acid, and auxin. Plant. Physiol..

[66] Gampala SS, Finkelstein RR, Sun SS, Rock CD (2002). ABI5 interacts with abscisic acid signaling effectors in rice protoplasts. J. Biol. Chem..

[67] Xie Y (2016). Arabidopsis HY1-Modulated Stomatal Movement: An Integrative Hub Is Functionally Associated with ABI4 in Dehydration-Induced ABA Responsiveness. Plant. Physiol..

[68] Mu Y (2017). BASIC PENTACYSTEINE Proteins Repress ABSCISIC ACID INSENSITIVE4 Expression via Direct Recruitment of the Polycomb-Repressive Complex 2 in Arabidopsis Root Development. Plant. Cell Physiol..

[69] Shkolnik-Inbar D, Bar-Zvi D (2010). ABI4 mediates abscisic acid and cytokinin inhibition of lateral root formation by reducing polar auxin transport in Arabidopsis. Plant. Cell.

[70] Tarnowski L (2020). Overexpression of the selective autophagy cargo receptor NBR1 modifies plant response to sulfur deficit. Cells.

[71] Zientara K (2009). Activity of the AtMRP3 promoter in transgenic Arabidopsis thaliana and Nicotiana tabacum plants is increased by cadmium, nickel, arsenic, cobalt and lead but not by zinc and iron. J. Biotechnol..

[72] Liu, N., Ding, Y., Fromm, M. & Avramova, Z. Endogenous ABA Extraction and Measurement from Arabidopsis Leaves. Bio Protoc 4 (2014). 10.21769/bioprotoc.1257 PMC4823005 27066522

[73] Dobrev PI, Hoyerova K, Petrasek J (2017). Analytical Determination of Auxins and Cytokinins. Methods Mol. Biol..

[74] Dobrev PI, Kaminek M (2002). Fast and efficient separation of cytokinins from auxin and abscisic acid and their purification using mixed-mode solid-phase extraction. J. Chromatogr. A.

[75] Martin K (2009). Transient expression in Nicotiana benthamiana fluorescent marker lines provides enhanced definition of protein localization, movement and interactions in planta. Plant. J..

[76] Shannon P (2003). Cytoscape: a software environment for integrated models of biomolecular interaction networks. Genome Res..

[77] Morris JH, Kuchinsky A, Ferrin TE, Pico A (2014). R. enhancedGraphics: a Cytoscape app for enhanced node graphics. F1000Res.

