# Supplementary material for: A selective autophagy cargo receptor NBR1 modulates abscisic acid signalling in Arabidopsis thaliana
Source: Sci Rep. 2020 May 8;10:7778. doi: 10.1038/s41598-020-64765-z (PMC7211012; doi:10.1038/s41598-020-64765-z)
Supplement: Supplementary file 1 — Supplementary Information. [file 41598_2020_64765_MOESM1_ESM.docx]

**Supplementary Material**

**A selective autophagy cargo receptor NBR1 modulates abscisic acid signalling in *Arabidopsis thaliana***

Tarnowski L, Collados Rodriguez M, Brzywczy J, Piecho-Kabacik M, Krčkova Z, Martinec J, Wawrzynska A, Sirko A

**List of Supplementary Material**

**Supplementary Figures:**

Fig. S1. Details of the transgenic lines obtained in this work.

Fig. S2. GO enrichment charts for different groups of OX DEGs.

Fig. S3. The HUBs enrichment in DEGs plotted against number of interactions with DEGs.

Fig. S4. Changes of expression of ABA-downregulated genes in shoots and roots of OX7.5, KO1 and KO3 in comparison to WT.

Fig. S5. Statistical analysis of the ABA, PA and DPA assay by LC-MS method.

Fig. S6. The level of ABA-related transcripts in fresh and stratified (cold-exposed after sowing) seeds in relation to the transcript level in the fresh seeds of WT plants.

Fig. S7. Statistical analysis of the results of measurement of ABA level in seeds.

Fig. S8. Statistical analysis of the stomata aperture in different lines without ABA treatment and treated with 10 μM ABA.

Fig. S9. Non-cropped blots used in Fig. 8B.

**Supplementary Tables:**

Tab. S1. List of differentially expressed genes (DEGs) in shoots of OX7.5.

Tab. S2. List of differentially expressed genes (DEGs) in roots of OX7.5.

Tab. S3. List of hubs having interaction with at least 5 DEGs in OX7.5 (top hubs).

Tab. S4. List of genes included in the enriched network shown in Fig. 3A (enriched network).

Tab. S5. List of ABA-related genes and their expression in OX and KO lines.

Tab. S6. Plant media composition.

Tab. S7. Oligonucleotides used as primers.

Tab. S8. Plasmids used in this study.

Tables S6-S8 are included in this file; Tables S1-S5 are submitted separately as a single Microsoft Excel (.xlsx) file.

**Supplementary Figures (Fig. S1-Fig. S8)**


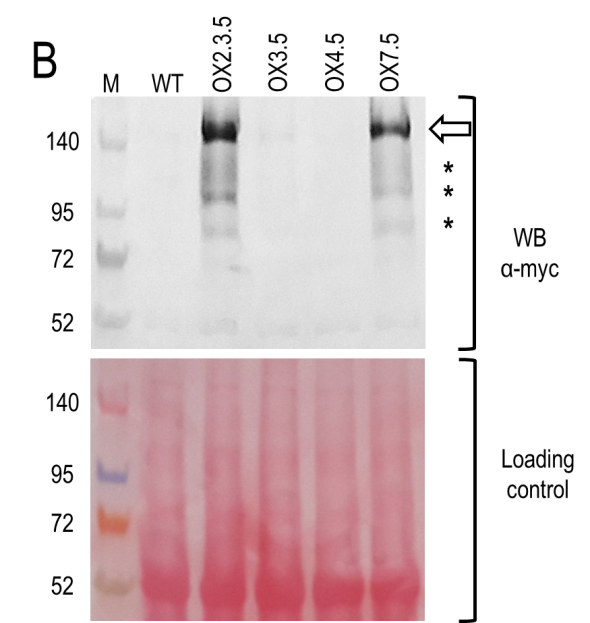

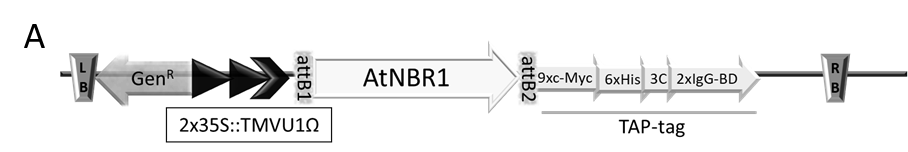


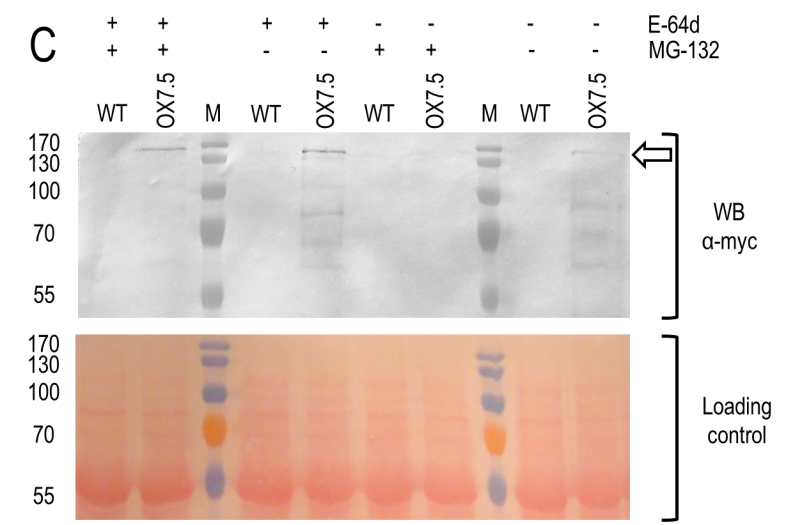


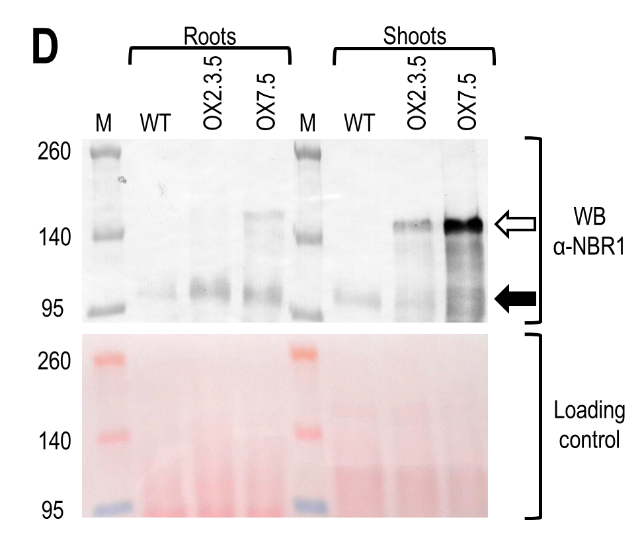


E


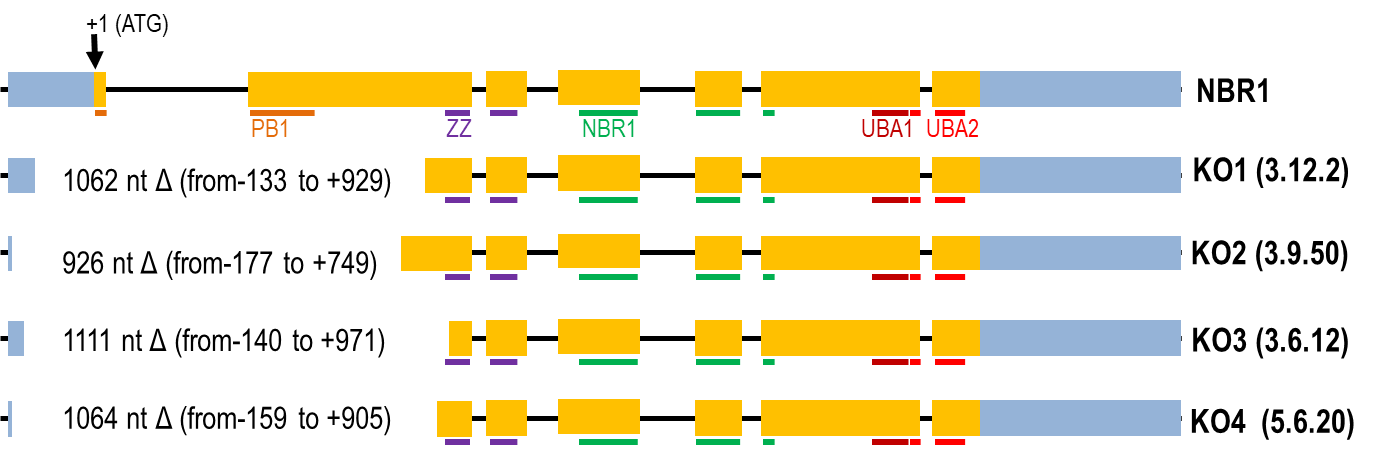


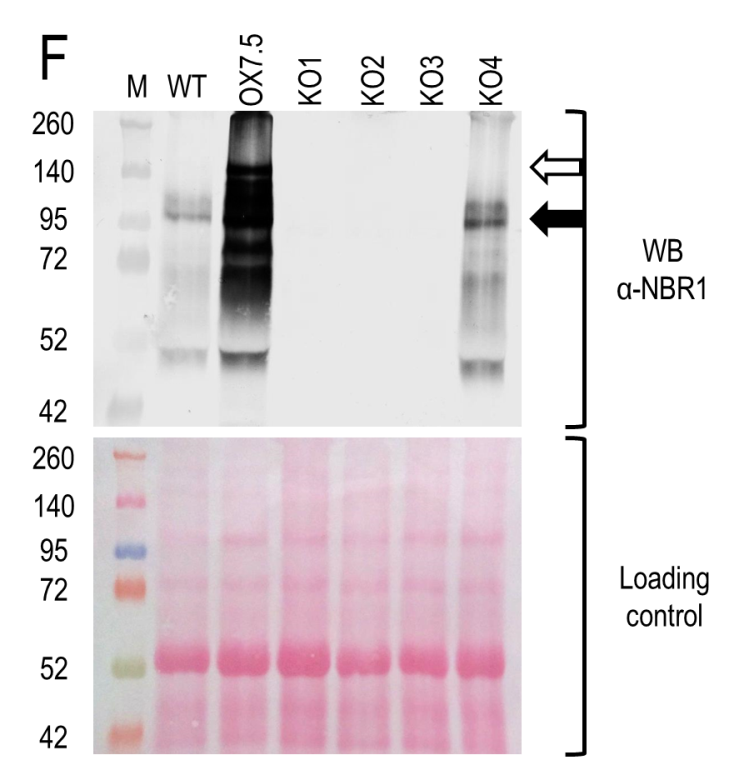


**Fig. S1. Details of the transgenic lines obtained in this work. (A)** Scheme of the T-DNA fragment present in the binary vector used to obtain the NBR1-OX (NBR1-TAP) lines. The vector elements are not drown to scale. AtNBR1, cDNA encoding the full length NBR1 without stop codon from *Arabidopsis thaliana*; GenR, gene coding for gentamycin resistance; LB, left border of T-DNA; RB, right border of T-DNA; attB1 and attB2, recombination sites; 2x35S::, two copies of the cauliflower mosaic virus promoter 35S; U1Ω, tobacco mosaic virus translational enhancer; TAP-tag, tandem-affinity purification tag composed of nine repetitions of the c-Myc epitope (9xc-Myc), 6 repetitions of His amino acid residue (6xHis), 3C protease cleavage site (3C) and two copies of the protein A IgG binding domain (2xIgG-BD). Nos ter, nopaline synthase terminator; T35S, terminator of the cauliflower mosaic virus. **(B)** The level of NBR1-TAP in 1-week-old seedlings. **(C)** Stability of NBR1-TAP in the presence of the autophagy inhibitor (E-64d) and the proteasome inhibitor (MG132). WB, western blot probed with the indicated primary antibodies; M, molecular marker. The loading control parts were stained with Ponceau S. The size of the marker (M) are indicated. NBR1-TAP and NBR1 are indicated by the white and black arrows, respectively. **(D)** The level of NBR1 in shoots and roots of 4-week-old plants grown hydroponically, determined by using anti-NBR1 antibodies. **(E)** The scheme of the deletions in nbr1-KO lines. yellow boxes, exons; blue boxes, UTRs, black lines, introns; the regions corresponding to the domains of NBR1 protein are indicated. **(F)** Example of the Western blot indicating the lack of NBR1 in the nbr1-KO lines. The nbr1-KO4 line, showing the presence of NBR1 is most probably heterozygous. It was discarded from the further analysis. Both the transgenic AtNBR1-TAP and the endogenous AtNBR1 proteins migrated slower than expected on sodium dodecyl sulphate-polyacrylamide gel electrophoresis (SDS-PAGE). The predicted sizes of AtNBR1-TAP and AtNBR1 were 110 kDa and 76 kDa, respectively, while their gel migration indicated sizes of about 140 kDa (and multiple bands below) and >100 kDa (double band), respectively. This apparent electrophoretic shift phenomenon of AtNBR1 was not investigated further experimentally; however, it suggested the possibility of posttranslational modifications, such as ubiquitination or sumoylation.

## Western blot analysis (Supplementary Method to Fig. S1)

For protein isolation, plant material (seedlings, leaves, roots) was homogenized in 100 μl extraction buffer (1M Tris-HCl, pH 8.0) with 1 μl Protein Inhibitor Cocktail (Sigma-Aldrich), and centrifuged at 13,000 rpm for 10 min at 4 °C. E64d (1μl from 10 mg/ml stock, APEBIO) and MG-132 (1 μl from 5 mM stock, Selleckchem) were added, if indicated. Proteins in the supernatant (50 µg/well) were separated on 8% SDS-PAGE gels, transferred to the nitrocellulose membrane (Bio-Rad), and visualized by staining with Ponceau S. Membranes were blocked with 5% non-fat dry milk and then probed with either mouse anti-Myc monoclonal IgG, or rabbit anti-AtNBR1 polyclonal IgG, generated against the N-terminal fragment of AtNBR1 consisting of 165 aa (custom-generated by GenScript), or anti-NBR1 (Agrisera, cat nr. AS142805A). Goat anti-mouse IgG (Sigma-Aldrich) and goat anti-rabbit IgG (Sigma-Aldrich) coupled to alkaline phosphatase were used as secondary antibodies, respectively. Protein bands were visualized by adding 5 mL 5-bromo-4-chloro-3-indolyl phosphate/nitro blue tetrazolium (BCIP/NBT) Solution (BioShop) at room temperature.


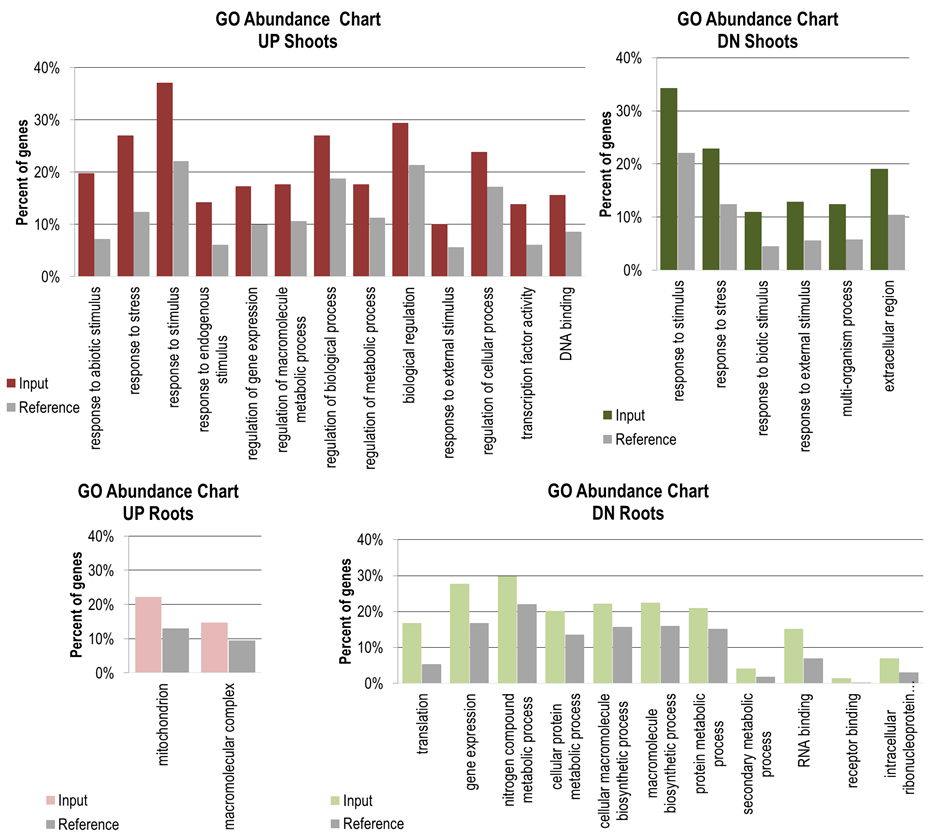


**Fig. S2. GO enrichment charts for different groups of OX DEGs.**

**Fig. S3. The HUBs enrichment in DEGs plotted against number of interacting DEGs.** Most enriched hubs are colored in red, pointed with arrows and labeled while remaining hubs are shown in grey.


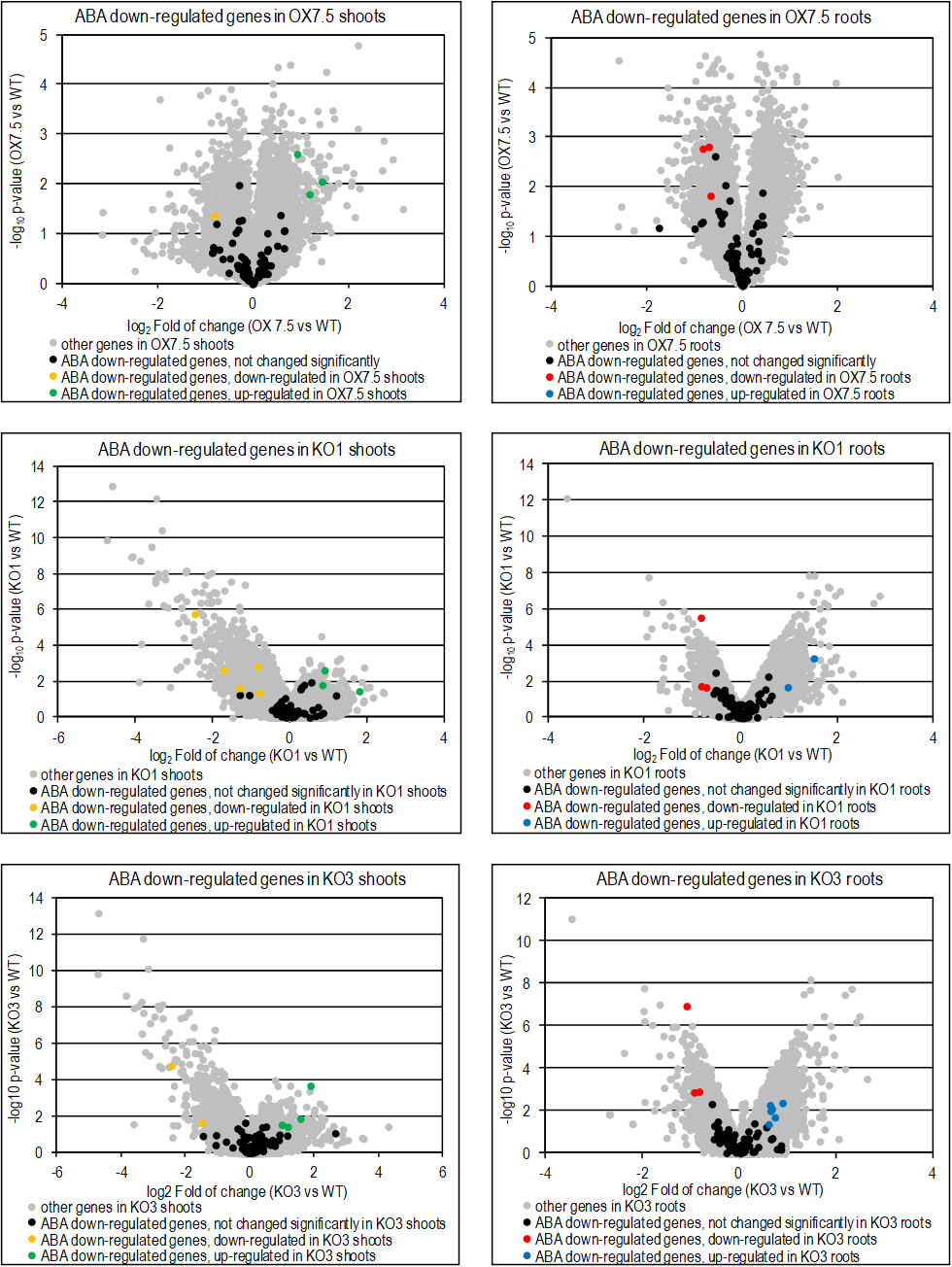


**Fig.S4.** **Changes of expression of ABA-downregulated genes in shoots and roots of OX7.5, KO1 and KO3 in comparison to WT.**

**Fig. S5. Statistical analysis of the ABA, PA and DPA assay by LC-MS method**.


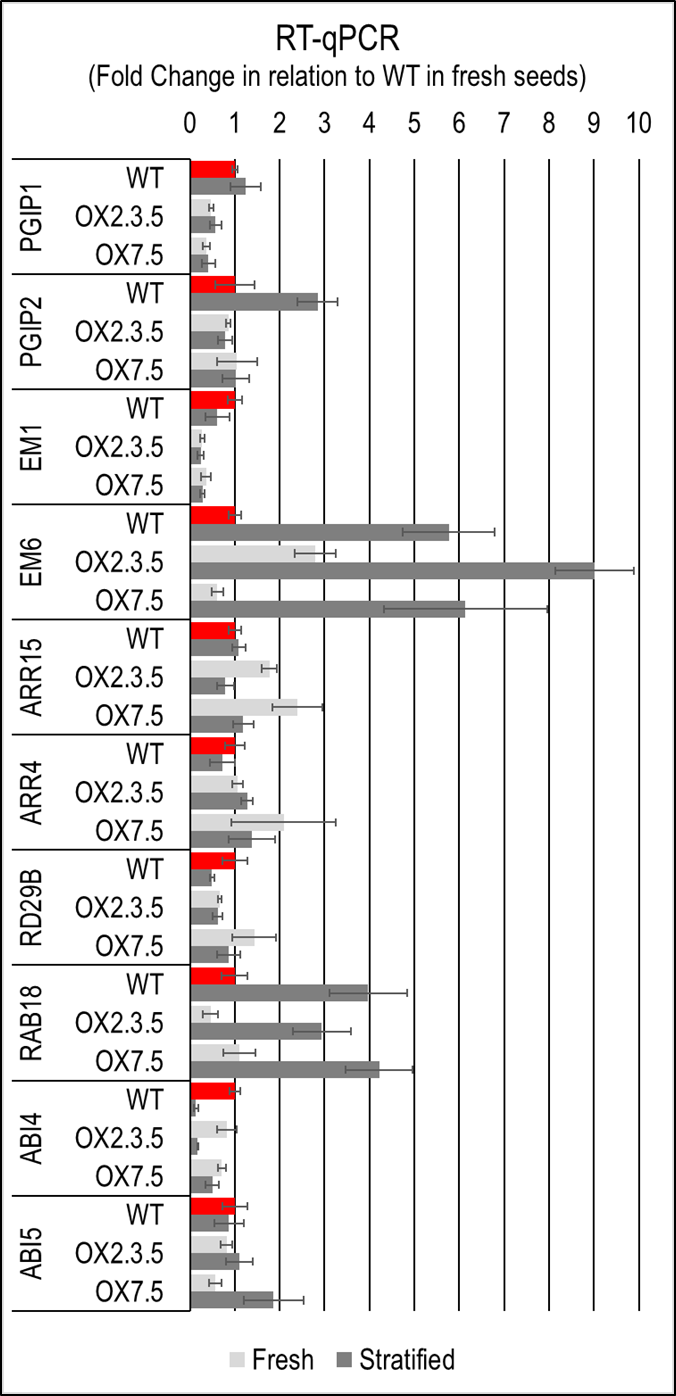


**Fig. S6. The level of ABA-related transcripts in fresh and stratified (cold-exposed after sowing) seeds in relation to the transcript level in the fresh seeds of WT plants.**

**Supplementary Fig. S7. Statistical analysis of the results of measurement of ABA level in seeds.**

**Fig. S8. Statistical analysis of the stomata aperture in different lines without ABA treatment and treated with 10 μM ABA.** The results of this test were used to indicate statistically significant differences in Fig. 3C.

**
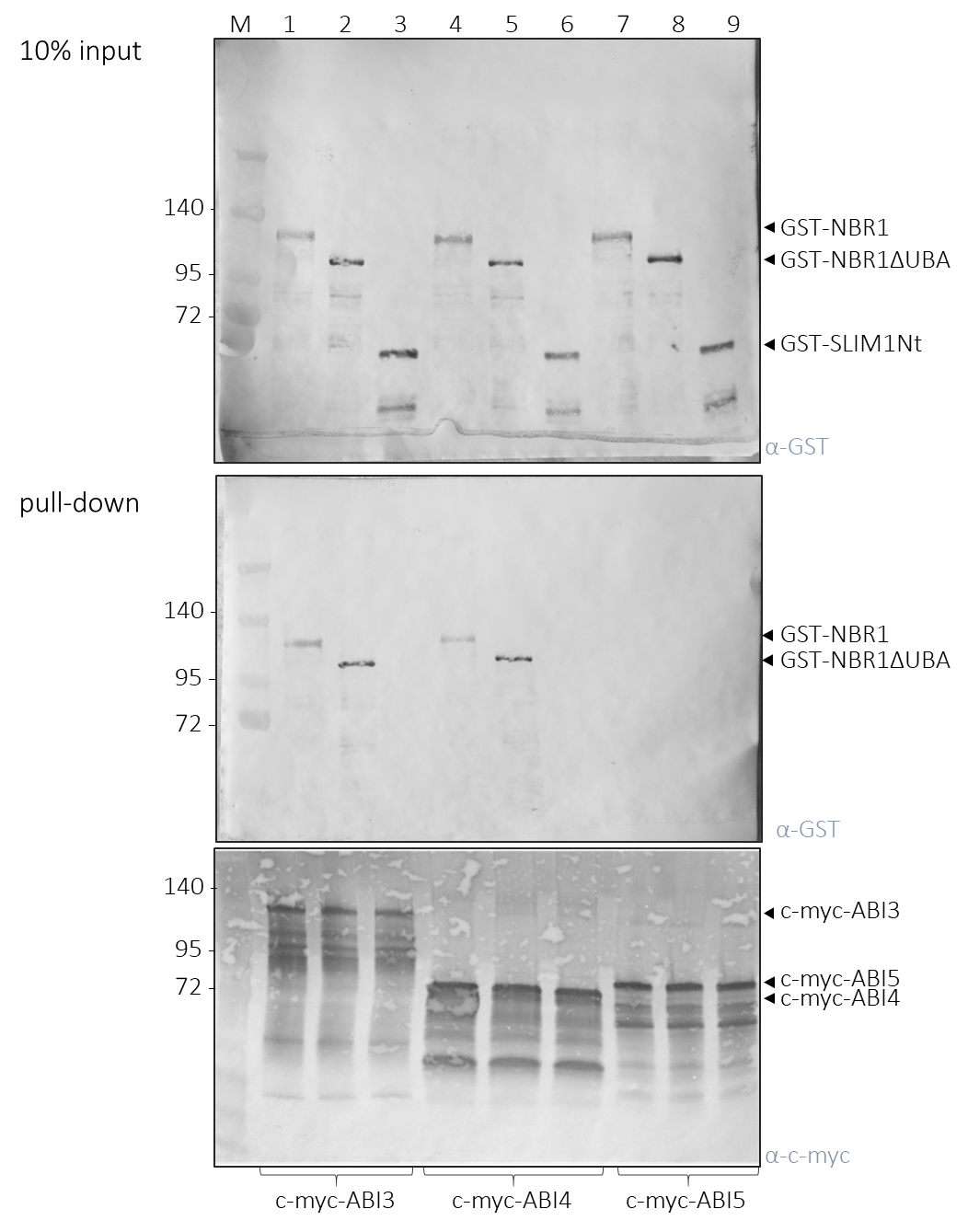
**

**Fig. S9.** Non-cropped blots used in Fig. 8B.

**Supplementary Tables (Tab. S1-S7)**

**Tab. S1**. List of differentially expressed genes (DEGs) in shoots of OX7.5 (in a separate file Suppl_Tables_S1-S5. xlsx).

**Tab. S2**. List of differentially expressed genes (DEGs) in roots of OX7.5 (in a separate file Suppl_Tables_S1-S5. xlsx).

**Tab. S3**. List of hubs having interaction with at least 5 DEGs in OX7.5 (top hubs) (in a separate file Suppl_Tables_S1-S5. xlsx).

**Tab. S4**. List of genes included in the enriched network shown in Fig. 3A (enriched network) (in a separate file Suppl_Tables_S1-S5. xlsx).

**Tab. S5.** List of ABA-related genes and their expression in OX and KO lines (in a separate file Suppl_Tables_S1-S5. xlsx).

**Tab. S6.** Plant media composition.

**Tab. S7.** Oligonucleotides used as primers.

**Tab. S8**. Plasmids used in this study.

**Tab. S6.** Plant media composition.

| **Salt** | **0.5 x modified Hoagland** | **0.5 x modified AB** [1] |
| --- | --- | --- |
| Macronutrients | final concentration (mM) | Final concentration (mM) |
| KNO_3_ | 3 | 1.75 |
| Ca(NO_3_)_2_x2H_2_O | 2 | 1.25 |
| NH_4_NO_3_ | - | 2.5 |
| NH_4_PO_4_ | 1 | - |
| K_2_HPO_4_ | 1 | 0.5 |
| MgSO_4_x7H_2_O | 1 | 0.5 |
| MgCl_2_ | 1 | 0.45 |
| CaCl_2_ | 1 | 1 |
| KCl | 1 | - |
| NaCl | - | 0.05 |
| Fe(Na)EDTA | 0.02 | 0.025 |
| Micronutrients | Final concentration (µM) | Final concentration (µM) |
| H_3_BO_3_ | 25 | 12.5 |
| Mn(NO_3_)_2_ | 2 | 5.0 |
| ZnC_4_H_6_O_4_ | 2 | - |
| Cu(NO_3_)_2_ | 0.1 | 0.32 |
| (NH_4_)_6_Mo_7_O_24_x4H_2_O | 0.1 | - |
| CoCl_2_x6H_2_O | - | 0.055 |
| Na_2_MoO_4_x2H_2_O | - | 0.41 |
| Zn(H_2_Cl_2_O_2_)_2_x2H_2_O | - | 0.048 |
| Buffered with 1mM MES | pH 5.5 | pH5.8 |

[1] Kulik et al. 2012. Plant Physiology 160: 868-83; DOI: 10.1104/pp.112.194472

**Tab. S7.** Oligonucleotides used as primers.

| Gene or fragment of gene/AGI | Purpose | Primer (Forward / Reverse) sequences (5’- 3’) |
| --- | --- | --- |
| PGIP1/At5g06860 | RT-qPCR (seeds) | AGTCCCTGACCTTCGCCTAT / TGGAGCTTGTTGCGGGATAA |
| PGIP2/At5g06870 | RT-qPCR (seeds) | GGAGTATTCCGGCTGAGTGG / TACTCTCCTTTGGGGATGCG |
| EM1/At3g51810 | RT-qPCR (seeds) | TGAGAAGGCGAAGCAAGGAG / CTCTTGAGCTTCGAGGCTGTG |
| EM6/At2g40170 | RT-qPCR (seeds) | ACGCAAAGGTGGTCTTAGCA / TCGTCTATCTCGACTCCTTCCT |
| ARR15/AT1G74890 | RT-qPCR (seeds) | GTGACGACTGTTGAGAGTGGG / ACCAGAAGATCCATTGTCTCCA |
| ARR4/AT1G10470 | RT-qPCR (seeds) | GAAACTCGCCGACGTGAAAC / GGTGGAGGAAGCGAAGAGTT |
| RD29B/AT5G52300 | RT-qPCR (seeds) | GGAGGGGAAAGGACATGGTG / ACTTCGGTTTACCACCGAGC |
| RAB18/AT5G66400 | RT-qPCR (seeds) | CTGAAGGCTTTGGAACTGGC / CGGTGAAGCATTCCTCCCAA |
| ABI4/AT2G40220 | RT-qPCR (seeds) | GGGCAGGAACAAGGAGGAAG / CCACCGAACCAGCTAGAGAG |
| ABI5/AT2G36270 | RT-qPCR (seeds) | AATAAGAGAGGGATAGCGAACG / GCTACCACCACCTCTATGTATC |
| Actin 2/AT3G18780 | RT-qPCR (reference gene) | GCACCAAGCAGCATGAAGAT / GGAACCACCGATCCAGACAC |
| NBR1/AT4G24690 | full length cDNA cloning | CACCATGGAGTCTACTGCTAAC / TCAAGCCTCCTTCTCCCCTGT |
| NBR1/AT4G24690 | Truncated (NBR1ΔUBA) cDNA cloning | CACCATGGAGTCTACTGCTAAC / TCATTCTATATCCTCCTGAAGAGGAAC |
| ABI3/AT3G24650 | Full length cDNA cloning | CACCATGAAAAGCTTGCATGTGGCG / TCATTTAACAGTTTGAGAAGT |
| ABI4/AT2G40220 | full length cDNA cloning | CACCATGGACCCTTTAGCTTCCCAA / TTAATAGAATTCCCCCAAGA |
| ABI5/AT2G36270 | full length cDNA cloning | CACCATGGTAACTAGAGAAACGAAG / TTAGAGTGGACAACTCGGGTT |
| NBR1/G2 (Guide-2) | CRISPR guide (gRNA) | ATT**GGAAGGAACATCCCTGCTCA** / AAACTGAGCAGGGATGTTCCTTCC |
| NBR1/G3 (Guide-3) | CRISPR guide (gRNA) | ATTG**AATTGAGAGCTGCCCTAGCT** / AAACAGCTAGGGCAGCTCTCAATT |
| NBR1/G144 (Guide-144) | CRISPR guide (gRNA) | ATTG**TTACGAGAAAAGCGGATTAC** / AAACGTAATCCGCTTTTCTCGTAA |

**Tab. S8**. Plasmids used in this study.

| **Plasmid name** | **Description or purpose** | **Reference** |
| --- | --- | --- |
| pENTR™ D/TOPO | Cloning CACC-PCR products | Invitrogen™ (Gateway^®^ Entry vector) |
| pENTRNBR1 | Cloning in Gateway system | This study |
| pENTRNBR1ΔUBA | Cloning in Gateway system | This study |
| pENTRABI4 | Cloning in Gateway system | This study |
| pENTRABI5 | Cloning in Gateway system | This study |
| pSITE-nEYFP-C1  (NYC) | Plant expression vector containing N terminal half of YFP (BiFC) | Chakrabarty et al. 2007. Mol Plant Microbe Interact. 20: 740; DOI: 10.1094/MPMI-20-7-0740 |
| pSITE-cEYFP-C1  (CYC) | Plant expression vector containing C terminal half of YFP (BiFC) | Chakrabarty et al. 2007. |
| pSITE-nEYFP-C1 NBR1  (NYC-NBR1) | Plant expression vector containing N terminal half of YFP (BiFC) in fusion with NBR1 | This study |
| pSITE-nEYFP-C1 NBR1ΔUBA  (NYC-NBR1ΔUBA) | Plant expression vector containing C terminal half of YFP (BiFC) in fusion with NBR1 without UBAs domains | This study |
| 35S:NBR1-YFP | AtNBR1 cloned into the plant expression vector pH7YWG2 | Lab stock |
| pSITE-cEYFP-C1-ABI3  (CYC-ABI3) | Plant expression vector containing C terminal half of YFP (BiFC) in fusion with ABI3 | This study |
| pSITE-cEYFP-C1-ABI4  (CYC-ABI4) | Plant expression vector containing C terminal half of YFP (BiFC) in fusion with ABI4 | This study |
| pSITE-cEYFP-C1-ABI5  (CYC-ABI5) | Plant expression vector containing C terminal half of YFP (BiFC) in fusion with ABI5 | This study |
| pYL436-AtNBR1-stop | Protein expression | This study |
| pDCH_G144G2 | Knock-out of NBR1 | This study |
| pDCH_G144G3 | Knock-out of NBR1 | This study |
| pDE_CAS9_G144G2 | Knock-out of NBR1 | This study |
| pDE_CAS9_G144G3 | Knock-out of NBR1 | This study |
| pDEST15 | Bacterial expression vector containing GST tag | Invitrogen™ |
| pDEST15_AW_MYC | Bacterial expression vector containing c-myc tag; based on pDEST15 vector but GST tag substituted with 10x c-myc tag | This study |
| pDEST15_NBR1 | Bacterial expression vector containing NBR1 with N-terminal GST tag | This study |
| pDEST15_NBRΔUBA | Bacterial expression vector containing NBR1ΔUBA with N-terminal GST tag | This study |
| pDEST15_SLIMNt | Bacterial expression vector containing N-terminal SLIM1 (At1g73730) with N-terminal GST tag | This study |
| pDEST15_AW_MYC_ABI3 | Bacterial expression vector containing ABI3 with N-terminal c-myc tag | This study |
| pDEST15_AW_MYC_ABI4 | Bacterial expression vector containing ABI4 with N-terminal c-myc tag | This study |
| pDEST15_AW_MYC_ABI5 | Bacterial expression vector containing ABI5 with N-terminal c-myc tag | This study |
